# Crafting Regional Identity and Peace through the ASEAN Way in Diplomacy

**DOI:** 10.12688/f1000research.179376.1

**Published:** 2026-04-27

**Authors:** Wildan Akbar Hashemi Rafsanjani, Herlina Juni Risma Saragih, Guntur Eko Saputro, Luhut Simbolon

**Affiliations:** 1 Universitas Pertahanan Indonesia, Bogor, Special Capital Region of West Java, Indonesia

**Keywords:** ASEAN, diplomacy, regional identity, peace, ASEAN way

## Abstract

The Association of Southeast Asian Nations (ASEAN) has developed a distinctive diplomatic approach known as the “ASEAN Way,” characterized by consensus-building, non-interference, and informal dialogue. This approach has played a significant role in shaping regional identity and maintaining peace in Southeast Asia, despite increasing geopolitical and transnational challenges. However, its effectiveness and adaptability in a rapidly changing global environment remain subjects of debate.

**Methods:**

This study adopts a qualitative research design based on the analysis of secondary data, including official ASEAN documents, academic literature, and case studies. A thematic analysis approach was employed to identify key patterns related to the principles of the ASEAN Way, its role in regional identity formation, and its contribution to peace and stability. Data triangulation and expert consultation were used to enhance the validity and reliability of the findings.

**Results:**

The findings indicate that the ASEAN Way has significantly contributed to regional peace by fostering mutual trust, dialogue, and conflict avoidance among member states. Its principles of consensus and non-interference have enabled cooperation across diverse political and cultural contexts, thereby supporting the development of a shared regional identity. However, the study also reveals limitations, particularly in addressing complex transnational challenges and responding effectively to urgent geopolitical issues due to its consensus-based decision-making process.

**Conclusions:**

The ASEAN Way remains a vital framework for regional diplomacy in Southeast Asia, promoting stability and cooperation. Nevertheless, to remain effective in the twenty-first century, ASEAN must adapt its traditional principles to address emerging challenges while maintaining its core values. Strengthening flexibility and responsiveness within the ASEAN Way is essential for sustaining regional peace and identity in an evolving global context.

## Introduction

Since its formation in 1967, the Association of Southeast Asian Nations (ASEAN) has established itself as a stable presence in the region and a key partner in regional cooperation. Established with the background of the Cold War situation and the local conflicts, ASEAN has expanded to become a strong ten-member unit that is an important factor in the politics, economy, and safety of the Asia-Pacific region ( Helen E. S. Nesadurai, 2008). One of the traits of ASEAN diplomacy is the ASEAN Way, which is a set of guidelines emphasizing reaching consensus, staying out of member states’ internal affairs, and having casual, non-confrontational conversations. The practice has played animportantpart in establishing a distinct regional identity and developing a feeling of belongingness to its multinational member states.

The ASEAN Way was developed as a result of the collective recognition of the Southeast Asian countries that togetherness and solidarity are keys to success in struggling with the many challenges that the region went through in the middle of the 20th century. The concepts of the ASEAN Way were developed in such a way that they could embrace a wide range of political structures, cultures, and historical backgrounds of its member states so that they could cooperate without violating their sovereignty ( Susy Tekunan, 2014). These principles have worked especially well to deter inter-state conflicts as well as to contain tensions in the region. Throughout the decades, ASEAN has been able to preserve peace and stability within the region, despite the region experiencing serious external forces and internal issues ( J. Ciorciari, 2017).

The consensus and non-interference that is highlighted by the ASEAN Way has both been strength and a weakness to the organization. On the one hand, these values have created the feeling of mutual trust and respect amongnations in the region, which has contributed to the region’s peace throughout history. It is also through the ASEAN Way that the entity has been capable of dealing with outside forces, striking a balance between relationships with the countries of China, the United States, and Japan, and making ASEAN a major force in both regional security and economy ( Rodon Pedrason, 2017). The reliance on consensus has, however, also raised its own criticisms, especially in cases where quick decision-making is needed. The ASEAN Way has been perceived to be too slow and cumbersome, particularly in light of upcoming security challenges and geopolitical changes ( Beverly Loke, 2005).

The South China Sea controversies provide a picture of the strengths and weaknesses of the ASEAN Way. Due to its consensus and non-intervention principles, ASEAN’s response to China’s claims to a large portion of the South China Sea has been limited and has resulted in a fragmented response among its member states ( Michael York, 2015). Although ASEAN has been in a position to preserve dialogue and avoid an outright conflict, its success in solving such disputes has been put in question. This has cast doubt on whether the ASEAN Way will be able to cope with the continually challenging and volatile security situation in Asia-Pacific ( M. Leifer, 1999).

Besides traditional security issues, ASEAN has gone further to tackle unconventional security problems, including environmental degradation, outbreak and pandemics, and transnational crimes. To advance the comprehensive strategy of regional security beyond the military problem, the principles of the ASEAN Way have been revised to permit regional coordination in a variety of spheres ( Heng, 2014). This adaptability has been crucial in ensuring that the ASEAN remains relevant in the rapidly evolving global environment, but it has also created new difficulties in upholding the principles of the ASEAN Way and striking a balance between the diverse interests of the member nations ( T. Husain, 2017).

This essay aims at discussing how the ASEAN Way can be employed to promote peace and regional identity in the Southeast Asian region. It will look at the historical background of the ASEAN Way, its influence on security and cooperation in the region, and how it has withstood the current geopolitical issues. It is with this analysis that the study seeks to offer a holistic perception of the effects of ASEAN Way on regional diplomacy and challenges it is experiencing in the twenty first century. This research paper will finally contribute to the overall debate of regionalism and international relations in Southeast Asia, which is a input on how the ASEAN can further evolve and continue to play the centre stage of the local order.

## Research methodology

The purpose of this paper, which is built on the qualitative research approach, is to comprehend how the ASEAN Way contributes to the development of provincial character and promotes peace in Southeast Asia. It is crucial to remember that qualitative research is especially appropriate for the current study since it will enable a thorough examination of the intricate social and diplomatic dynamics that quantitative studies are unable to fully capture ( Akbar Hashemi Rafsanjani et al., 2026). Qualitative research, as Creswell (2014) observes, is also good at giving a comprehensive picture of the situation and the involved processes in the interactions between people, which makes it the best choice to analyse the delicate dynamics of the diplomatic work of ASEAN. Furthermore, this method enables academics to interpret diplomatic norms, institutional practices, and regional cooperation patterns in a real-world environment. It also promotes a thorough grasp of how ASEAN member nations develop shared values and collective identity through conversation and consensus-building diplomacy.

It analyses it using several sources of data, which include case studies, academic literature, and official ASEAN documents. The primary data sources may be joint statements, the Treaty of Amity and Cooperation (TAC), the ASEAN Charter and other official forms. These readings can be instrumental in clarifying the official ideas and principles according to which the ASEAN Way works and how it is implemented in the regional diplomatic practice. Furthermore, the scholarly sources are instructive and offer perspectives and analyses carried out by experts in the field of study that supplement the understanding of how the ASEAN Way affects regional identity and peace. Furthermore, scholarly sources are informative and offer insights and analyses that have been carried out by experts in the research area, which supplement the knowledge on the effects of the ASEAN Way on regional identity and peace. The most significant sources will be the works of such authors as Susy Tekunan (2014), Beverly Loke (2005), and Helen E. S. Nesadurai (2008), who have researched the ASEAN diplomatic structure and its functions in the sphere of regional security in detail.

The process of data collection was conducted by conducting a review of the literature and a critical analysis of the available documents. This method is consistent with the recommendations suggested by Creswell (2014) and Sugiyono (2018), who consider the necessity to triangulate data obtained through different sources to increase the reliability and validity of the qualitative research. This study seeks to offer a full analysis of the ASEAN Way by analysing the primary documentation as well as secondary sources. In addition; past scholarly publications on ASEAN diplomacy and regional cooperation were reviewed to discover common themes and theoretical approaches. This technique enables the study to synthesize findings from a variety of scholarly sources, resulting in a thorough knowledge of the ASEAN Way in regional government.

They are analysed using thematic analysis, which is a popular method in qualitative research, enabling the detection and explanation of patterns in the figures ( Braun and Clarke, 2006). Key subjects are identified concerning the principles of non-interference, consensus decision-making, and how it affects the peace and identity of the region, using this method. In this case, thematic analysis comes in especially handy because it can be employed to reveal the latent meanings and implications of the ASEAN practices in diplomacy.

In order to achieve the strength and dependability of the answers, a number of measures were put inplace. As advised by Sugiyono (2018), data triangulation was used to check the information in various documents and literature. As well, peer debriefing and consultation with people in the Southeast Asian studies were also done to offer critiques and ensure that interpretations of the data were factual and sound. The practice of reflexivity was also applied, in which a researcher kept on reflecting about his/her biases and how they could affect the interpretation of data ( Creswell, 2014).

Although this study had a very rigorous methodology, this research has various limitations. Main aspects of these include reliance on secondary data material, which might not completely reflect the dynamic and changing nature of the ASEAN diplomacy. Moreover, the results of the present research can be restrictive in terms of generalizability, as it was done in the context of ASEAN and due to the peculiarities of Southeast Asia. According to Sugiyono (2018), a qualitative study is always time and place-specific, and its results tend to be local to the location of the research.

The qualitative research design that was used in the current study offers a strong platform through which the ASEAN Way and its impact on the identity of the region and peace can be explored. This research allows us to have a comprehensive picture of how the ASEAN diplomatic practice has resulted in the stability and cohesion of southeast Asia by integrating a combination of document analysis, literature and thematic analysis. The methodological rigor of the research takes into account the limitations of qualitative research, i.e., the possibility of triangulating the results and consulting the experts, ensuring the credibility of the results obtained.

## Result and discussion

### Definition and concept of the ASEAN way

The ASEAN Way, a major trademark of the ASEAN is a distinct diplomatic and procedural approach that the association has employed since its inception in 1967. The agreement-making, absence of interference of the domestic affairs of the member states and unofficial diplomacy are all believed to be the pillars of the Southeast Asian dispute resolution and cooperation in the region. These ideals emphasize the importance of consultation and respect amongst member states so that ASEAN is able to solve the issues relating to the region by diplomacy and not by conflict. Subsequently, the ASEAN Way has been considered as a significant instrument in facilitating stability and trust among the various political regimes of the Southeast Asia ( Buszynski, 1997).

The necessity to manage the diverse political, economic, and cultural orientations of the ASEAN member states and foster the growth of regional cooperation gave rise to the ASEAN Way. Beverly Loke (2005) suggests that the ASEAN Way which is characteristic of most cultural treatment of the management of international security that is instilled in the minds of ASEAN policy makers, sums up the behavioral and procedural forms which have been bureaucratized within ASEAN to thwart and handle conflicts. Although this strategy has been achieved through various challenges caused by the conflicting national or national interests as well as external pressure, it has played a significant role in the preservation of peace and security in the region.

One of the key tenets of the ASEAN Way is non-interference, which member nations have rigorously adhered to. This rule guarantees every country sovereignty and autonomy in its domestic affairs, which also contributes to the respect and trust of the ASEAN members for one another ( Tekunan, 2014). The principle was originally developed to prevent conflicts and decrease tensions amongst newly independent Southeast Asian republics. It also contributes to diplomatic unity by encouraging communication and consultation instead of direct engagement in domestic political affairs. The non-interference principle is regarded as an asset and a drawback of the ASEAN Way. Even though it has been significant in safeguarding peace among the member states, it has been faulted on failing to take more decisive action on those issues that would require collective action such as the violation of human rights and threats across national boundaries.

The concept of the ASEAN Way is also highly connected with the radical and historical situation of Southeast Asia. Katsumata (2003) argues that the ASEAN Way was formulated due to the interest of the Southeast Asian countries in redefining international standards, such as non-interference in the well-defined environment in the region. Political issues that have had an impact on this restoration project are the urge to preserve domestic peace and the issue of state sovereignty. These issues gave birth to the ASEAN Way which is perceived as a viable solution to regional politics which relies on the principles of autonomy and non-interference as the means of protection against external invasion and internal turmoil.

The ASEAN Way has not been without its problems, particularly when it came to attempting to adjust to new geopolitical and security demands to do so despite its anchor value in the diplomacy culture of ASEAN. In one instance, Haacke (1999) notes that the concept of flexing engagement by some ASEAN members is an in-house problem to the ASEAN Way that suggests a more activist-oriented approach to the region, which may require the assessment of the rigorous compliance. This debate emerged as ASEAN was facing increasing regional challenges such as political crisis and transnational security challenges which required concerted efforts. It also suggests the increasing awareness that there might be a need to change the conventional diplomatic practices by ASEAN to remain relevant in the changing regional environment. This elicits a dilemma in adhering to the set standards and new realities to conform, thus depicting the dynamic characteristics of the ASEAN Way and its capacity to evolve. This discussion arose when ASEAN confronted growing regional difficulties, including political crises and transnational security concerns, which necessitated collective action. It indicates a growing realization that ASEAN’s traditional diplomatic tactics may need to evolve in order to stay effective in a changing regional environment. This highlights the conflict between adhering to established norms and adapting to new realities, demonstrating the dynamic nature of the ASEAN Way and its capacity for evolution.

Additionally, the ASEAN Way has been crucial in helping ASEAN take the lead in regional security cooperation, particularly through the ASEAN Defence Ministers Meeting (ADMM) and ASEAN Regional Forum (ARF). These platforms, which are based on the principles of the ASEAN Way, have made it possible for ASEAN to engage the main external powers without diminishing its importance in the regional security framework ( Heng, 2014). The ASEAN Way has also played an instrumental role in assisting ASEAN to be at the forefront in cooperating on regional security, especially ASEAN Defence Ministers Meeting (ADMM) and ASEAN Regional Forum (ARF). It is through these platforms that are founded on the principles of the ASEAN Way that the ASEAN has been enabled to involve the primary external powers without downgrading its role in the regional security structure ( Heng, 2014). Nonetheless, these engagements are inherently constrained by the same norms via which these engagements are executed, especially, consensus-based decision-making process, which is prone to deliver stalling and watered-down conclusiveness during crisis periods.

It is true that ASEAN has succeeded in ensuring peace and security in Southeast Asia due to its own approach to regional diplomacy, which is called the ASEAN Way. The ASEAN Way is facing challenge due to the necessity to adjust to the new regional and global processes despite its success in promoting the development of collaboration and avoiding conflicts among the member states. As ASEAN strives to overcome them, the ASEAN Way will probably remain extolled as a source of peace as much as it will be criticized to fail in meeting more and more complicated and global issues.

Another important aspect of the ASEAN Way is that it focuses on communication and consensus as some of the key regional cooperation tools. Rather than formal legal mechanisms or force, ASEAN is promoting amongst member countries consultation and creating a gradual trust. This is the diplomacy that has helped the region to be stable, even with the various political systems, cultures, and historical challenges. Leszek Buszynski (1997) argues that the cooperative approach of ASEAN has helped the member states to settle differences between each other without necessarily engaging in a dispute. Equally, Juergen Haacke (1999) notes that the ASEAN Way depicts a common diplomatic culture which appreciates harmony, mutual respect, joint effort among the regions when addressing political and security issues.

### ASEAN Regional identity

A regional identity in ASEAN is a complex and emerging phenomenon, a peculiarity of which is dictated by a strange mix of the past, cultural diversity, and political expediency. During the formation of the ASEAN in 1967, it has been struggling to make sure that the member states share a common identity that supersedes the massive differences within the member states. The ASEAN Way’s tenets of non-interference, consensus-building, and respect for sovereignty are fundamental to this identity.

The ASEAN regional identity has been established through the principles of common norms and values that are meant to promote peace and stability in Southeast Asia. Rumelili (2007) has indicated that ASEAN has managed to promote the development of a collective identity of Southeast Asian states on the basis of these norms that have led to a high degree of conflict among a group of diverse states with a history of conflict. Such a sense of collective identity is not just a creation of unified cultural and historical experiences but also a political instrument that is aimed at strengthening corporate cooperation and integration in the region.

The history of the development of the regional identity in ASEAN can be dated back to the period when the major agenda of this organization was to temper inter-state relations and to avoid conflict. According to Nesadurai (2008), the initial emphasis ASEAN had was to stabilize the inter-state relations, which provided the foundation of wider regional identity. This identity has, over the years, expanded to include security cooperation as well as economic and socio-cultural integration, which can be seen through such missions as the ASEAN Community and ASEAN Charter.

The adoption of the Charter of ASEAN in 2008 is a great stepping stone in the process of forming the regional identity of ASEAN. It codifies the values and principles guiding the ASEAN Community and preconditions the further integration in many aspects, among which are politics, security, and economics ( Lin, 2010). The adoption of the Charter is the way ASEAN commits itself to a rules-based organization, which respects its collaborative norms in the face of globalization and regionalization.

Irrespective of these attempts, the process of creating a regional identity in ASEAN has not been free of obstacles. Cultural diversity of the member states of ASEAN, language, political systems, etc, is a major challenge facing the establishment of a strong regional identity. According to Koh (2007), the attempts of ASEAN to establish a socio-cultural community and ASEAN identity have to be balanced between the establishment of the regional identity and the acknowledgement of individual identities of the member states. This strike is essential in the process of making sure that the regional integration process does not undermine the cultural and political peculiarities that distinguish each member state.

Moreover, the capability of ASEAN as a regional identity is also doubtful when it comes to its relations with outside forces and its contribution to the overall East Asian regionalism. Though ASEAN has gone further towards the establishment of a common regional identity, as it is contended by Jones and Smith (2007), the reality of international relations and the role of external players still weigh down on the cohesiveness of this identity. As had been the case with the ASEAN plus Three (APT) process, there are complexities of extending the regional identity of ASEAN to the Northeast Asian nations, such as China, Japan, and South Korea, whereby divergent interests and identities are involved ( Terada, 2003).

The regional identity of ASEAN is a dynamic and multifaceted construct that has been at the core of the activities of the organization in ensuring that the region of Southeast Asia is at peace, stable, and cooperative. Although there has been a lot of progress concerning the promotion of a collective identity, concerns related to internal diversity and the external forces seem to remind ASEAN that it should always evolve and enhance its regional identity. The ASEAN Charter and the ASEAN Civic efforts are some of these steps in this direction, but it is the capacity of ASEAN to reconcile the alterations in the interests of the member states with the overall objective of regional integration that will determine whether these efforts will succeed.

Table 1 describes the institutional procedures employed by the ASEAN diplomacy to maintain peace. Member states participate in communication, preventative diplomacy, and cooperation via forums such as the ASEAN Summit, the ASEAN Regional Forum (ARF), and the ASEAN Political-Security Community (APSC). These procedures minimize tensions, resolve conflicts peacefully, and strengthen stability in Southeast Asia, illustrating how the ASEAN Way promotes long-term regional peace.

**Table 1.** ASEAN Institutional mechanisms supporting regional peace.

ASEAN mechanism	Function	Contribution to regional peace
ARF	Stage for discourse on safetymatters	Promotes confidence-building among regional actors
APSC	Framework for radical and security cooperation	Encourages collective approaches to regional stability
TAC	Establishes principles for peaceful relations	Institutionalizes peaceful conflict resolution
ASEAN Summit	Annual meeting of ASEAN leaders	Strengthens policy coordination and regional unity
ASEAN Defence Ministers’ Meeting (ADMM)	Dialogue among defenseministers	Enhances military cooperation and security collaboration

**Figure 1.**
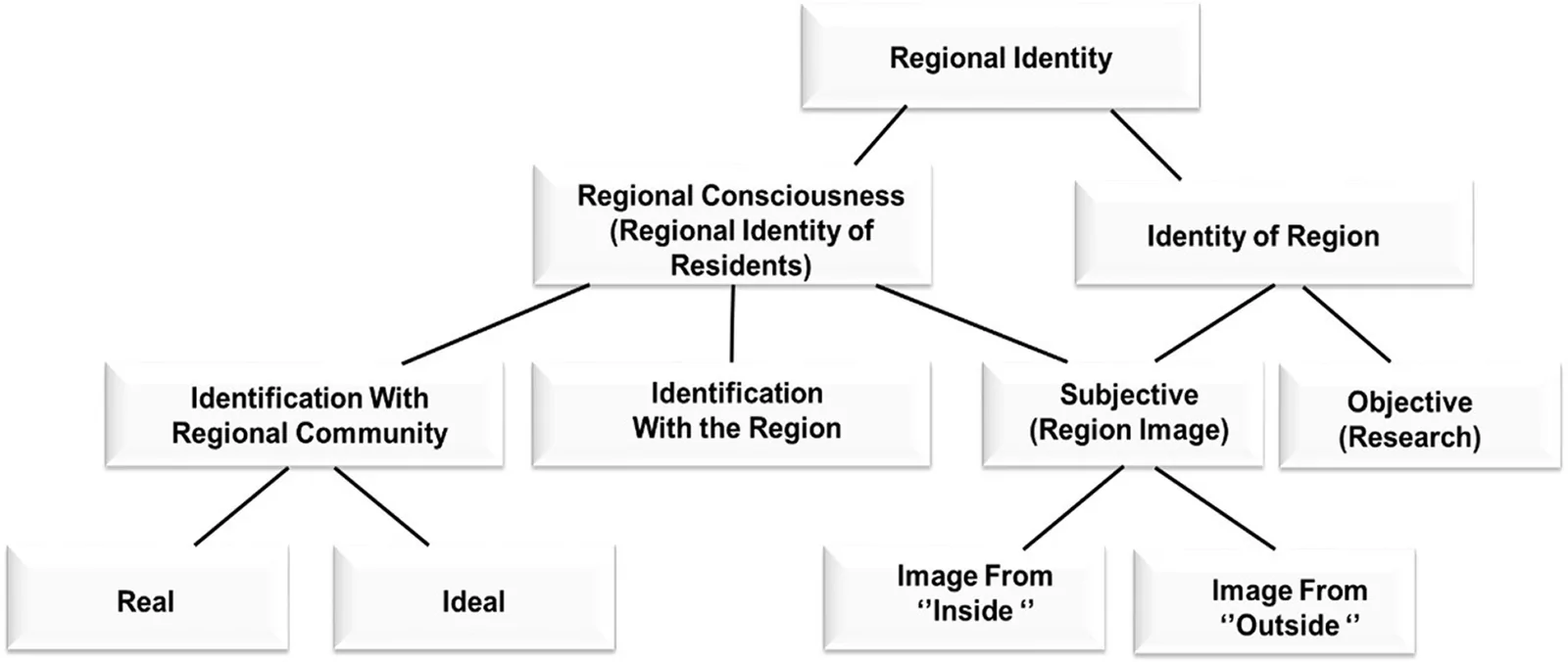
Conceptual framework of regional identity formation.

Figure 1 demonstrates the concept of the regional security and diplomatic organization of ASEAN which connects the member states of ASEAN and other foreign countries. It highlights the fact that ASEAN is the most important diplomatic center in the Asia-Pacific region. Through the ASEAN regional forum (ARF), ASEAN plus Three (APT) and the East Asia Summit (EAS) institutional forums, ASEAN is assisting the conversation and collaboration with relevant regional and global communities including China, Japan, South Korea and the US, among others. These models encourage proactive diplomacy, dispute resolution, and confidence-building. As a result, ASEAN uses diplomatic communication and multilateral cooperation to bring about peace and stability in the area Paasi (1986).

### The role of the ASEAN way in regional peace

The so-called ASEAN Way, which avoids the use of force, consensus-building, and casual mediation, has been judged essential to Southeast Asia’s peace and security. In spite of the complicated political and cultural environment of the area, where it was founded in 1967, ASEAN has proven to be one of the most positive partners of the region in terms of conflict management and, subsequently, the advancement of the law and order.

The ASEAN peace is intensely embedded in the ideals of the organization especially the principle of non-interference. Since every state considers the sovereignty of other states and does not interfere with their internal affairs, this principle has turned out to be very crucial in avoiding conflicts among the ASEAN member states. According to Tekunan (2014), the principle of non-intervention that exists in the ASEAN Way has created an environment of mutual respect and trust between its countries of the region, which has played an essential role in ensuring peace in the region. This has enabled ASEAN to move through the high tensions without necessarily engaging in conflict which ensures that the peace in the region is maintained.

Moreover, consensus based decision-making has been one of the primary reasons, which has simultaneously resulted in the absence of conflicts and improved coordination among the member states of the ASEAN Way due to its heterogeneous composition. Beverly Loke (2005) argues that despite some of the situations where such a strategy may lead to delay in decision-making, it has been good to ensure that the member states feel included and their concerns are heard. This participatory style has helped in preventing worsening of conflict situations since decisions are made when there is a broad agreement among the members hence reducing probability of action being pursued on personal terms which can lead to conflict.

Another way the ASEAN Way has contributed to peace in the region is how ASEAN has been managing external war and other security issues. As an example, the reaction of ASEAN to the Cambodian crisis in the late 1970s and 1980s explains the success of the ASEAN Way in addressing regional security problems. Kyudeug Hwang (2019) explains the way ASEAN, by applying the principles of ASEAN Way, managed to contribute to the solution to the Cambodian conflict in a substantial manner, thus cementing its peace and stability agenda in Southeast Asia. The success is highly referred to as a prime example of how ASEAN can control the security in the region with its own diplomatic system.

But the ASEAN Way does not pass without criticism. Other scholars opine that it is the same principles that make the ASEAN Way a successful mechanism that can restrict its ability to counter more nuanced, transnational security threats like non-interference and consensus. Heng (2014) notes that the ASEAN Way has had some success in ensuring peace in the region; however, it encounters many challenges when it comes to external issues like the aggressiveness of China in the South China Sea. The consensus form of approach can cause inactivity or poor reactions, especially when member states have conflicting interests or when outside forces are highly influential.

Despite these obstacles, the ASEAN Way has remained essential to maintaining peace in Southeast Asia. Despite being at the center of the regional security structural composition, its principles have allowed ASEAN to include the major external powers in regional security debates, notably the ASEAN Regional Forum (ARF). As Husain (2017) argues, the fact that ASEAN has been able to promote the positive and negative peace, i.e. to minimize the occurrence of direct conflict and increase the levels of cooperative relationships, proves that ASEAN Way remains topical and applicable in the context of the changing security environment of Southeast Asia.

The ASEAN Way has played a crucial role in promoting and maintaining peace in the region of Southeast Asia. Due to the non-interference, consensus, and informal diplomacy principles, ASEAN has been in a position to prevent conflicts in the region, facilitate cooperation, and solve complex security problems. Although the shortcomings of the ASEAN Way still exist within the framework of emerging problems and transnational threats, it is still a significant paradigm in the cycle of providing peace and security to Southeast Asia.

As seen in Table 2, ASEAN diplomatic rules build up a similar identity among member states. The principles of agreement, non-interference and informal diplomacy as exemplified in the ASEAN Way promote a culture of cooperation in politics and countries with divergent political systems and cultures can work together. This saw ASEAN gradually forge a regional identity based on trust, dialogue and respect.

**Table 2.** Core principles of the ASEAN way and their role in regional identity formation.

ASEAN way principle	Description	Contribution to regional identity
Consensus-based decision making	Decisions are made through agreement among all member states rather than voting.	Promotes unity and collective ownership of regional policies
Non-interference	Member states avoid intervening in the internal affairs of others	Builds mutual respect and trust among diverse political systems
Informal diplomacy	Preference for dialogue, consultation, and informal negotiation	Encourages cooperative culture and long-term diplomatic relationships
Quiet diplomacy	Disputes are handled through behind-the-scenes negotiations	Prevents escalation of conflicts and preserves regional harmony
Respect for sovereignty	Each state’s political independence and territorial integrity are recognized.	Strengthens shared identity while respecting national diversity

**Figure 2.**
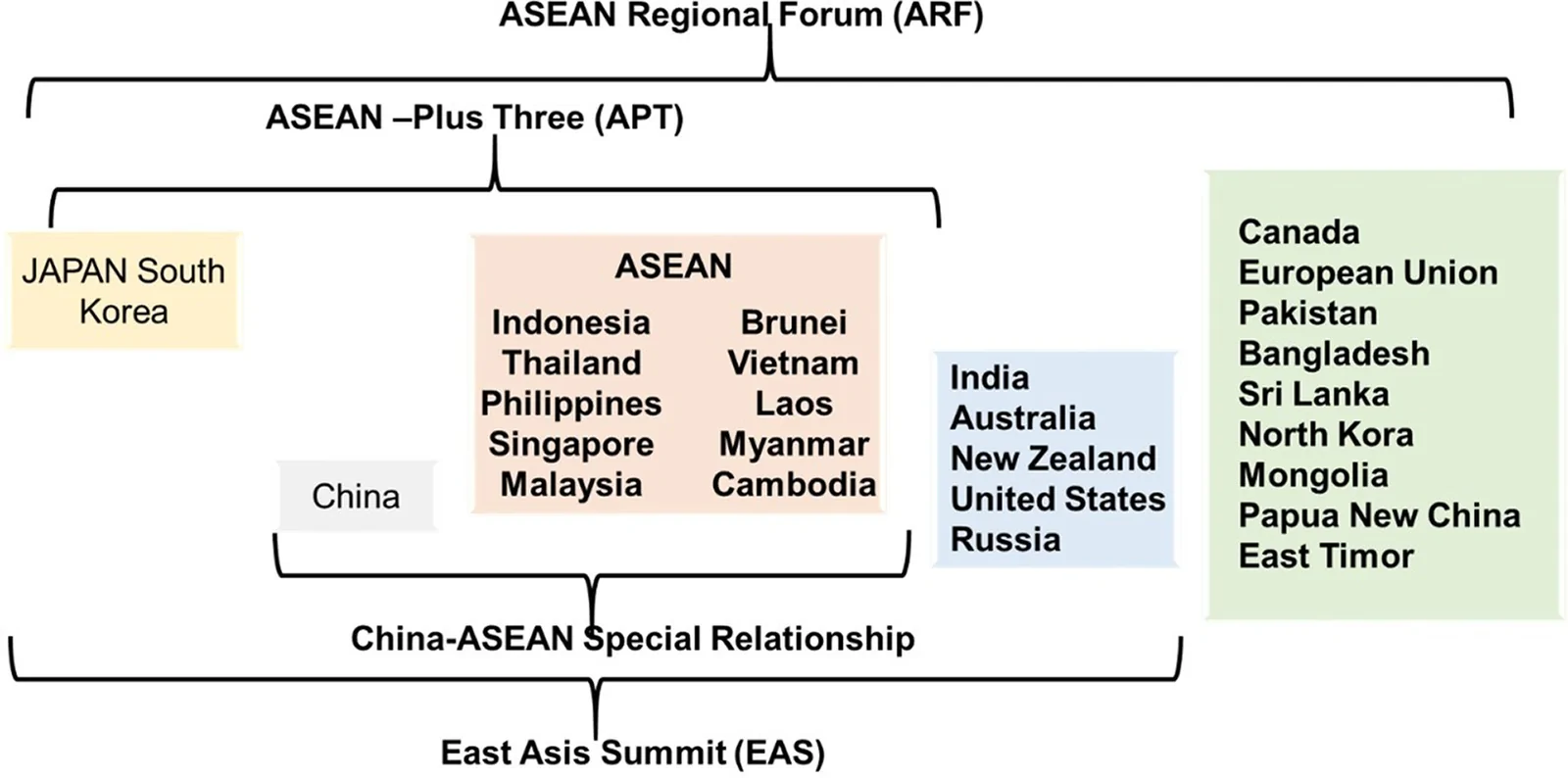
Structure of ASEAN-centered regional security and diplomatic forums.

Figure 2 shows the conceptual framework for regional identity building inside a regional organization, such as ASEAN. According to the theory, regional identity comes from the interaction of two fundamental dimensions: regional consciousness and the region’s identity. Regional awareness refers to how individuals, groups, and states understand themselves as part of a larger regional community. In contrast, the region’s identity describes how the territory is characterized by both internal perceptions (“image from inside”) and external perspectives (“image from outside”). These interwoven factors help to build a collective sense of belonging, shared standards, and common values, all of which contribute to the creation of a stronger regional identity ( Sokla, 2019).

### Challenges to the ASEAN way

Over the past 50 years, ASEAN has been successful in maintaining peace and fostering collaboration in Southeast Asia thanks in large part to consensus, non-interference, and informal diplomacy the cornerstones of the ASEAN Way. This special arrangement has seen ASEAN scale through the extensive and intricate political, cultural, and economic environment of the region to become one of the most successful regional corporations beyond Europe.

Nevertheless, with the ongoing development of the region and the world, the ASEAN Way has to struggle with great challenges. The values that used to have guaranteed unity and stability are now viewed as a limitation to dealing with contemporary, transnational challenges that are manifested in the form of human rights, economic integration, and non-traditional security dangers. The consensus principle can contribute to inefficiency and slowness, and the principle of non-interference usually delays the ASEAN capability to efficiently handle internal crises inside member states. Since the beginning, learning have tended to focus on the obstacles facing the application of universal human rights norms in the region ( Aguirre and Pietropaoli, 2012).

These challenges notwithstanding, the ASEAN Way continues to be a key tool of regional diplomacy in Southeast Asia. Its flexibility and focus on communication and collaboration are still useful in dealing with the complex procedures of the region. But to survive and remain pertinent in the twenty-first century, ASEAN can have to reconsider its initial concepts to become more decisive and acting as one without acting in opposition to the interest of its member states. ASEAN follows the concept of non-interference both in their political and economic sphere. This time it faced trouble as well. Jong, K. (2010). ASEAN Free Trade Area (AFTA) and ASEAN Surveillance Program (ASP) can be further integrated to integrate regional economies.

Finding a balance between the core values that have historically formed the basis of the ASEAN success and adjusting to the requirements of a more interconnected and complex global context is likely to become the main staple of the future of the ASEAN Way. In so doing, the ASEAN will remain on the frontline of achieving prosperity, peace and stability in the Southeast Asian region and beyond.

## Conclusion

Over the past 50 years, ASEAN has successfully ensured peace and cooperative efforts in South East Asia due to the ASEAN Way, which is based on the principles of consensus, non-interference, and informal diplomacy. This distinctive strategy has enabled ASEAN to pass through the complicated and multicultural political, cultural, and economic environment of the area and be one of the most successful regional entities outside of Europe.

Nevertheless, ASEAN Way encounters very serious challenges as the world and the region are evolving. The ideals that previously ensured unity and stability are now seen as constraints in the quest to achieve modern global issues such as human rights of the human being, economic integration as well as the unconventional security threats. The reliance on consensus may result in inefficiencies and delayed responses, even if the concept of non-interference may frequently restrict ASEAN’s capacity to handle domestic problems within member nations.

These challenges notwithstanding, The ASEAN Way has remained a key model in the diplomacy of the Southeast Asian region. Its flexibility and emphasis on communication and cooperation remain useful in managing the complexity of the region. Nevertheless, to remain relevant and effective in the twenty-first century, ASEAN may have to alter its approach to the situation, perhaps by redefining its principles to allow it to take more decisive steps and be more integrated without compromising the diverse interests of its member states.

The capacity of the ASEAN Way to balance both the need to maintain the main principles underpinning the success of ASEAN and the adaptation to the requirements of a more interconnected and complex globalized environment is likely to define the survival of the ASEAN Way. This way, ASEAN is able to retain its central position in promoting prosperity, peace and security in southeast Asia and beyond.

## Limitations of the study

Despite the fact that this piece of work makes good contributions to the development of knowledge on how the ASEAN Way can contribute to the establishment of regional identity and regional peacekeeping in Southeast Asia, there are several limitations that should be taken into consideration and could influence the depth of the conclusions and generalizability. Firstly, the research paper relies on the secondary sources of data, including academic literature, official sources of the ASEAN, and case studies. Although these sources are valuable in terms of qualitative information, they might not be a comprehensive reflection of the latest trends and subtle views of the important stakeholders in ASEAN. Moreover, the use of publicly available documents might restrict the level of analysis, especially in the internal dynamics and decision-making process in ASEAN.

Second, the emphasis on qualitative analysis, though suitable to study the intricate social and political phenomena, is intrinsically associated with the inability to measure the effect of the ASEAN Way on the regional performance. This qualitative design offers a detailed approach but does not give a chance of statistical generalization as would have been the case in a mixed methods study, which would use quantitative data.

Third, the study focuses on a conceptual and historical analysis of the ASEAN Way and its significance for peace and regional identity. As a result, when applied to specific and contemporary circumstances, it does not fully address the real-life problems and responses of the member nations in the ASEAN manner. Future research can be more empirical in nature, involving interviews with officials and practitioners, in order to gain firsthand knowledge of the difficulties and efficacy of the ASEAN Way in the current geopolitical situation.

Lastly, the paper also has limitations in terms of its geographical scope because it is based in Southeast Asia, and this is not entirely representative of the external factors and global trends that are increasingly affecting the region. Although the paper also brings up the issue of potential external actors (China and the US) and the impact they have on the ASEAN Way, a further discussion of the impact of global geopolitical changes on the ASEAN Way can yield a more comprehensive view of whether it is viable or not.

To sum up, although this study is a valuable addition to the scholarly discussion on ASEAN and its peculiarities of the regional diplomacy, these shortcomings indicate that future research that will fill these voids, possibly due to a more varied methodology and a wider geographic focus, is necessary.

## Data Availability

Figshare: Crafting Regional Identity and Peace through the ASEAN Way in Diplomacy. https://doi.org/10.6084/m9.figshare.31797496.v1 [ Akbar Hashemi Rafsanjani et al., 2026]. The project contains the following underlying data: Qualitative dataset (ASEAN diplomatic documents and thematic analysis codes used in the study). Data are available under the terms of the Creative Commons Attribution 4.0 International license (CC-BY 4.0).
