# Crafting Regional Identity and Peace through the ASEAN Way in Diplomacy

**DOI:** 10.12688/f1000research.179376.3

**Published:** 2026-08-14

**Authors:** Wildan Akbar Hashemi Rafsanjani, Herlina Juni Risma Saragih, Guntur Eko Saputro, Luhut Simbolon

**Affiliations:** 1 Universitas Pertahanan Indonesia, Bogor, Special Capital Region of West Java, Indonesia

**Keywords:** ASEAN, diplomacy, regional identity, peace, ASEAN way

## Abstract

The Association of Southeast Asian Nations (ASEAN) has developed a distinctive diplomatic approach known as the “ASEAN Way,” characterized by consensus-building, non-interference, and informal dialogue. This approach has played a significant role in shaping regional identity and maintaining peace in Southeast Asia, despite increasing geopolitical and transnational challenges. However, its effectiveness and adaptability in a rapidly changing global environment remain subjects of debate.

**Methods:**

This study adopts a qualitative research design based on the analysis of secondary data, including official ASEAN documents, academic literature, and case studies. A thematic analysis approach was employed to identify key patterns related to the principles of the ASEAN Way, its role in regional identity formation, and its contribution to peace and stability. Data triangulation and informal peer debriefing were used to enhance the validity and reliability of the findings.

**Results:**

The findings indicate that the ASEAN Way has significantly contributed to regional peace by fostering mutual trust, dialogue, and conflict avoidance among member states. Its principles of consensus and non-interference have enabled cooperation across diverse political and cultural contexts, thereby supporting the development of a shared regional identity. However, the study also reveals limitations, particularly in addressing complex transnational challenges and responding effectively to urgent geopolitical issues due to its consensus-based decision-making process.

**Conclusions:**

The ASEAN Way remains a vital framework for regional diplomacy in Southeast Asia, promoting stability and cooperation. Nevertheless, to remain effective in the twenty-first century, ASEAN must adapt its traditional principles to address emerging challenges while maintaining its core values. Strengthening flexibility and responsiveness within the ASEAN Way is essential for sustaining regional peace and identity in an evolving global context.

## Introduction

Since its formation in 1967, the Association of Southeast Asian Nations (ASEAN) has established itself as a stable presence in the region and a key partner in regional cooperation. Established with the background of the Cold War situation and the local conflicts, ASEAN has expanded to become a strong ten-member unit that is an important factor in the politics, economy, and safety of the Asia-Pacific region ( Helen E. S. Nesadurai, 2008). One of the traits of ASEAN diplomacy is the ASEAN Way, which is a set of guidelines emphasizing reaching consensus, staying out of member states’ internal affairs, and having casual, non-confrontational conversations. The practice has played an important part in establishing a distinct regional identity and developing a feeling of belongingness to its multinational member states.

The ASEAN Way was developed as a result of the collective recognition of the Southeast Asian countries that togetherness and solidarity are keys to success in struggling with the many challenges that the region went through in the middle of the 20th century. The concepts of the ASEAN Way were developed in such a way that they could embrace a wide range of political structures, cultures, and historical backgrounds of its member states so that they could cooperate without violating their sovereignty ( Susy Tekunan, 2014). These principles have worked especially well to deter inter-state conflicts as well as to contain tensions in the region. Throughout the decades, ASEAN has been able to preserve peace and stability within the region, despite the region experiencing serious external forces and internal issues ( J. Ciorciari, 2017).

The theoretical foundation of this research is constructivism, which suggests that normative, ideational, and identity factors have greater effects on international affairs than material factors. From this perspective, ASEAN diplomacy is not only an instrument-based interaction but also a socially constructed behavior that is affected by the ASEAN Way, emphasizing cooperation, non-interference, and informal communication. Gradually, states in ASEAN construct a common regional identity based on mutual respect and peace through repeated diplomatic interactions and practices. Thus, institution-based norms shape the behavior of states and help to construct the regional order of ASEAN, and vice versa.

Constructivism is used here in a specific rather than a general sense, and the mechanism assumed by this study runs in three steps. Repeated meetings, consultations and joint declarations first make particular ways of speaking and behaving routine among the officials who take part in them. Those routines then harden into expectations that member states hold of one another. Finally, the expectations raise the political cost of acting against them, even though no legal penalty attaches to doing so. Identity on this account is a by-product of habit rather than of sentiment, and it affects state behaviour through reputation rather than through enforcement. It is also necessary to separate three outcomes that are often merged in writing on ASEAN. Preventing war between member states, managing disagreement so that it does not escalate, and building a community whose members no longer treat force as a live option are three different achievements, and the absence of war in Southeast Asia does not by itself demonstrate the third. Regime survival interests, deepening trade dependence, the shared experience of external threat during the Cold War, and ordinary strategic calculation may account for a good deal of the peace observed. This study therefore treats the ASEAN Way as one contributing factor among several rather than as the sole explanation for regional peace.

## Literature review

The consensus and non-interference that is highlighted by the ASEAN Way has both been strength and a weakness to the organization. On the one hand, these values have created the feeling of mutual trust and respect among nations in the region, which has contributed to the region’s peace throughout history. It is also through the ASEAN Way that the entity has been capable of dealing with outside forces, striking a balance between relationships with the countries of China, the United States, and Japan, and making ASEAN a major force in both regional security and economy ( Rodon Pedrason, 2017). The reliance on consensus has, however, also raised its own criticisms, especially in cases where quick decision-making is needed. The ASEAN Way has been perceived to be too slow and cumbersome, particularly in light of upcoming security challenges and geopolitical changes ( Beverly Loke, 2005).

The South China Sea controversies provide a picture of the strengths and weaknesses of the ASEAN Way. Due to its consensus and non-intervention principles, ASEAN’s response to China’s claims to a large portion of the South China Sea has been limited and has resulted in a fragmented response among its member states ( Michael York, 2015). Although ASEAN has been in a position to preserve dialogue and avoid an outright conflict, its success in solving such disputes has been put in question. This has cast doubt on whether the ASEAN Way will be able to cope with the continually challenging and volatile security situation in Asia-Pacific ( M. Leifer, 1999). The territorial disputes among ASEAN member countries such as Vietnam, the Philippines, Malaysia, Brunei, and China regarding the South China Sea are important sources of geopolitical instability in Southeast Asia. The dispute became aggravated by China’s nine-dash line claim, which overlapped with the zones recognized by UNCLOS, as well as its island construction and military expansion activities. The US responded to the conflict by ensuring free passage of sea and challenging the exaggerations with Freedom of Navigation Operations (FONOPs). ASEAN’s reaction to the issue, however, is fragmented owing to its internal disagreements and decision-making process known as the ASEAN Way.

Dermawan et al. (2024) uses a qualitative methodology based on interviews, documentation, online research, and archival studies to investigate the ability and competence of Indonesian regional administrations to carry out parallel diplomacy. The findings show that unequal budget distribution and officials’ lack of regulatory expertise lead to unequal regional diplomatic capacity. However, the study’s limited scope to Indonesian regional contexts limits its applicability in other contexts. In the research, the researcher ( Ajis et al., 2026) employs the historical analytical method to investigate the relations between Malaysia and Indonesia in terms of migration. In particular, based on data collected from policy and historical documents, the researcher analyses the problems of irregular migration and migration diplomacy based on Memoranda of Understanding (MOUs). The findings prove that both countries have always managed to settle their disagreements through cooperation and communication based on close cultural and historical relations even if sometimes migration causes some tensions for the relations of these two countries. Utomo (2025) examines Surabaya’s paradiplomacy (2016–2020) in promoting overall sustainability through international city collaborations, especially with Liverpool and Kitakyushu. Although it is limited by decentralization constraints, it uses qualitative analysis of policy papers and partnerships to identify better SDG integration. The research emphasizes the significance of subnational diplomacy in global engagement and urban environmental governance.

These sub-national studies matter to the present argument for a particular reason. Utomo (2025) shows Surabaya building direct links with Liverpool and Kitakyushu without working through Jakarta or the ASEAN Secretariat, and Dermawan et al. (2024) show that Indonesian regions differ widely in their capacity to do the same. Read against the regional level, this cuts both ways. City partnerships extend regional engagement into areas such as urban environment, waste management and public services that ASEAN's intergovernmental machinery has been slow to reach, and to that extent they support the regional project. At the same time they create working relationships that bypass ASEAN institutions altogether, and where capacity is uneven they widen the distance between well-resourced and poorer localities. Paradiplomacy therefore adds to the volume of regional engagement while weakening ASEAN's claim to be the region's single diplomatic channel, and it is this double effect that connects the sub-national literature to the questions about regional identity raised later in this paper.

Besides traditional security issues, ASEAN has gone further to tackle unconventional security problems, including environmental degradation, outbreak and pandemics, and transnational crimes. To advance the comprehensive strategy of regional security beyond the military problem, the principles of the ASEAN Way have been revised to permit regional coordination in a variety of spheres ( Heng, 2014). This adaptability has been crucial in ensuring that the ASEAN remains relevant in the rapidly evolving global environment, but it has also created new difficulties in upholding the principles of the ASEAN Way and striking a balance between the diverse interests of the member nations ( T. Husain, 2017).

In particular, with the assistance of the ASEAN Humanitarian Assistance Center and the ASEAN Intergovernmental Commission on Human Rights (AICHR), the framework of human rights in ASEAN has gradually transformed through both institutional changes and informality within ASEAN ( Maulana & Putra, 2024). The balance between the long-standing ASEAN values of consensus and non-interference on one hand. Though the ASEAN Way constrains ASEAN institutions, ASEAN has continually transformed the institutions governing its region to address transboundary haze pollution ( Charusombat, 2023). ASEAN has enhanced regional cooperation and policy coordination for ecological governance through gradual institution transformation, including new projects, actors, and interpretation of the ASEAN Agreement on Transboundary Haze Pollution (AATHP).

The Asia-Pacific area has gradually moved toward more formal institutions that are more precise and structured ( Suzuki, 2024). These changes show how local governance processes are changing, with nations adopting more adaptable governing structures while preserving their sovereignty and taking into account a variety of national interests within intricate regional arrangements.

To address transnational issues in the digital era, ASEAN has incrementally enhanced regional cooperation through diplomatic and legal means. The progressive move by ASEAN towards regional governance, which is still characterized by international cooperation and principles of sovereignty, can be observed through such measures as the ASEAN Mutual Legal Assistance Treaty (AMLAT). Due to the varied nature of the circumstances in which the member nations exist, regional integration in ASEAN requires structural change and national policy measures ( Prirayani, 2025). The findings reveal that a more adaptable approach to governance and institutional cooperation is necessary due to the heterogeneous nature of regional policy measures and their inability to help ASEAN integration.

This essay aims at discussing how the ASEAN Way can be employed to promote peace and regional identity in the Southeast Asian region. It will look at the historical background of the ASEAN Way, its influence on security and cooperation in the region, and how it has withstood the current geopolitical issues. It is with this analysis that the study seeks to offer a holistic perception of the effects of ASEAN Way on regional diplomacy and challenges it is experiencing in the twenty first century. This research paper will finally contribute to the overall debate of regionalism and international relations in Southeast Asia, which is a contribution to the debate on how ASEAN can further evolve and continue to play the centre stage of the local order.

The ASEAN Way’s rigorous devotion to consensus and non-interference norms presents serious institutional challenges notwithstanding its benefit to regional stability. The ability of ASEAN institutions to enforce regional decisions or step in during political and humanitarian crises is limited because they primarily function through intergovernmental collaboration rather than global authority. Because of this, rather than using legally binding regulations, ASEAN’s institutional structures frequently rely on voluntary adherence and diplomatic persuasion. ASEAN’s capacity to effectively address pressing regional issues including human rights abuses and internal conflicts has come under fire as a result of these institutional limitations.

The issue of Rohingya refugees has shed light on the weaknesses of the ASEAN human rights regime’s institutions. Because of some restrictions in the Terms of Reference (TOR) and because of ASEAN’s commitment to principles of consensus and non-intervention, Limsiritong (2018) states that “the potential for the ASEAN Inter-Governmental Human Rights Commission (AICHR) to effectively promote and protect human rights is limited.” The consultative nature of the AICHR renders it unable to take any firm steps when faced with critical humanitarian crises such as the Rohingya issue, notwithstanding the establishment of AICHR under Article 14 of the ASEAN Charter for the promotion and protection of human rights.

However, there are concerns about the efficiency of the application of ASEAN’s usual practices in addressing legal and political matters in cases of civil unrest, especially as seen in the case of Myanmar. In their article, Limsiritong and Sookhakich (2023) assert that the Myanmar crisis is likely to raise more criticisms of the ASEAN Way due to the lack of clear definition of ‘serious breach’ in the ASEAN charter, and the continued adherence to consultative and consensus approaches to decision-making. The article continues to explain that there is limited interpretive ability by the ASEAN Secretariat. Also, Article 5(3) will depend on political consensus at the ASEAN Summit for its interpretation. These limitations show how ASEAN’s ability to successfully address serious social and political crises can be weakened by the norms of agreement and non-interference.

Whereas Utomo (2025) explores city-based Para diplomacy toward sustainability, Ajis et al. (2026) look at the bilateral diplomacy of migration between Malaysia and Indonesia, and where Dermawan et al. (2024) focus on sub-national Para diplomacy capacity in Indonesia, all three studies are institutionally and empirically confined to sub-national actors or state-state relations without linking them to the framework of ASEAN regional governance institutions. In taking an innovative perspective of integrating Para diplomacy, bilateral migration diplomacy, and sub-national governance perspectives in analyzing ASEAN level regional politics, the uniqueness of the study lies in the effect of diplomatic norms on ASEAN Way.

Several further works help place these arguments on firmer ground. Acharya (2014) sets out criteria for judging whether consultation, socialisation, restraint and repeated interaction have produced a security community, and those criteria are used in this paper as a test rather than as an assumption. Ba (2009) treats regional identity as something negotiated and contested over time, which fits the uneven record described above better than a view in which identity follows automatically from membership. Stubbs (2019) presents the case made by ASEAN sceptics alongside that of its supporters, and his distinction between institutional durability and actual problem-solving is one this study adopts. Davies (2013) identifies a gap between what ASEAN states commit to on paper and what they do in practice, which bears directly on the human rights record discussed below. Jackson (2014) explains why states in the region hedge rather than align fully with a single major power, and Hu (2023) offers a close assessment of what ASEAN has and has not achieved in the South China Sea. Taken together, these studies caution against counting continued dialogue as successful dispute management.

Evidence drawn from other regions is used in this paper only for comparison, and not as direct evidence about ASEAN. Bani Salameh and Alotaibi (2026) show how Kuwait has built influence through neutrality, mediation and diplomatic credibility despite limited material power, which parallels the informal and preventive diplomacy on which ASEAN relies. Bani Salameh and Darabseh (2026) trace how the Gulf crisis reshaped Qatari-Turkish relations while weakening Gulf Cooperation Council cohesion, a pattern that resembles ASEAN’s divisions over China and the South China Sea. Salameh and Mubiadeen (2025) find that small Gulf states combine neutrality, hedging and alliance diversification in different proportions, which helps explain why ASEAN members can share regional norms while pursuing very different national security policies. Bani Salameh (2023) connects domestic vulnerability, regional cooperation and outside partnerships in the case of Bahrain, and Bani Salameh (2024) shows how Kuwait maintains working relations with rival powers without committing to either. Both cases offer a useful comparison with ASEAN members that trade heavily with China while relying on the United States for security.

## Research methodology

The purpose of this paper, which is built on the qualitative research approach, is to comprehend how the ASEAN Way contributes to the development of regional identity and promotes peace in Southeast Asia. It is crucial to remember that qualitative research is especially appropriate for the current study since it will enable a thorough examination of the intricate social and diplomatic dynamics that quantitative studies are unable to fully capture ( Akbar Hashemi Rafsanjani et al., 2026). Qualitative research, as Creswell (2014) observes, is also good at giving a comprehensive picture of the situation and the involved processes in the interactions between people, which makes it the best choice to analyse the delicate dynamics of the diplomatic work of ASEAN. Furthermore, this method enables academics to interpret diplomatic norms, institutional practices, and regional cooperation patterns in a real-world environment. It also promotes a thorough grasp of how ASEAN member nations develop shared values and collective identity through conversation and consensus-building diplomacy.

It analyses it using several sources of data, which include case studies, academic literature, and official ASEAN documents. The primary data sources may be joint statements, the Treaty of Amity and Cooperation (TAC), the ASEAN Charter and other official forms. These readings can be instrumental in clarifying the official ideas and principles according to which the ASEAN Way works and how it is implemented in the regional diplomatic practice. Furthermore, the scholarly sources are instructive and offer perspectives and analyses carried out by experts in the field of study that supplement the understanding of how the ASEAN Way affects regional identity and peace. Furthermore, scholarly sources are informative and offer insights and analyses that have been carried out by experts in the research area, which supplement the knowledge on the effects of the ASEAN Way on regional identity and peace. The most significant sources will be the works of such authors as Susy Tekunan (2014), Beverly Loke (2005), and Helen E. S. Nesadurai (2008), who have researched the ASEAN diplomatic structure and its functions in the sphere of regional security in detail.

Two terms need to be fixed before the analysis proceeds, since they are used in different senses in the wider literature. Peace is used in this study in the narrow sense of the absence of armed conflict between member states, together with the continued use of diplomatic channels when disputes arise. It does not refer to domestic stability, to human security, or to positive peace in the fuller sense, and no claim is made in this paper about those outcomes. Regional identity is treated as observable rather than assumed, and is traced through four indicators: the recurrence of a common vocabulary across official ASEAN statements; formal commitments entered into through ASEAN instruments; convergence in the positions member states take in regional forums; and the habit among officials of describing problems as regional rather than as purely national. Where the evidence supports only some of these indicators, that is stated in the results rather than passed over.

### Criteria for document selection and data sources

The official ASEAN articles were chosen based on their significance for regional identity creation, institutional governance, peacebuilding, and diplomacy. Documents that explicitly addressed ASEAN ideals, regional cooperation, conflict resolution, or institutional procedures associated with the ASEAN Way were included. The ASEAN Charter, Treaty of Amity and Cooperation (TAC), ASEAN Political-Security Community (APSC) Blueprint, ASEAN Summit joint statements, ASEAN Regional Forum (ARF) declarations, and official reports on regional security and humanitarian issues were among the important documents examined in this study. Additionally, scholarly works published between 1999 and 2026 were chosen for their applicability to discussions of regional integration, human rights governance, ASEAN diplomacy, and institutional transformation.

In total, forty-two official ASEAN documents published between 1976 and 2025 were examined. These comprised the ASEAN Charter, the Treaty of Amity and Cooperation and its three protocols, the ASEAN Political-Security Community Blueprint, twenty-six Summit and Foreign Ministers' joint communiques, and eight ASEAN Regional Forum chairman's statements. A document was included if it was issued in ASEAN's name, was publicly available in English, and dealt with regional order, dispute settlement, membership or institutional procedure. Bilateral agreements between individual member states, purely economic instruments with no security or normative content, and internal working papers not released publicly were excluded. Alongside these primary sources, sixty-one journal articles and books published between 1997 and 2026 were used, selected on the basis of peer review and direct relevance to ASEAN diplomacy, regional identity, human rights governance or institutional change. Triangulation was carried out by checking each thematic claim against at least one primary ASEAN document and one independent scholarly source, and any claim resting on only one of the two is identified as such in the results.

The process of data collection was conducted by conducting a review of the literature and a critical analysis of the available documents. This method is consistent with the recommendations suggested by Creswell (2014) and Sugiyono (2018), who consider the necessity to triangulate data obtained through different sources to increase the reliability and validity of the qualitative research. This study seeks to offer a full analysis of the ASEAN Way by analysing the primary documentation as well as secondary sources. In addition; past scholarly publications on ASEAN diplomacy and regional cooperation were reviewed to discover common themes and theoretical approaches. This technique enables the study to synthesize findings from a variety of scholarly sources, resulting in a thorough knowledge of the ASEAN Way in regional government.

They are analysed using thematic analysis, which is a popular method in qualitative research, enabling the detection and explanation of patterns in the figures ( Braun and Clarke, 2006). Key subjects are identified concerning the principles of non-interference, consensus decision-making, and how it affects the peace and identity of the region, using this method. In this case, thematic analysis comes in especially handy because it can be employed to reveal the latent meanings and implications of the ASEAN practices in diplomacy.

To identify recurring themes regarding ASEAN diplomacy and governance, all selected articles and scholarly sources underwent rigorous reading and review. Themes like decision-making processes based on consensus, non-interference, regional identity, institutional constraints, conflict resolution, and regional cooperation were identified through open coding. With the purpose of exploring the way ASEAN Way promotes regional identity building and peace, those codes were grouped under broader thematic categories. The identified themes were conceptualized via comparative study of secondary scholarly sources and primary documents from ASEAN.

Coding proceeded in two rounds. In the first round each document was read line by line, and passages were tagged wherever they referred to how decisions are made, how disagreement is handled, or how the region is described as a unit. The theme of regional consciousness, to take the clearest example, was built from three specific markers: the use of collective terms such as “we in the region” or “the ASEAN family” in place of individual state names; the description of a problem as shared rather than as belonging to one member; and appeals to ASEAN precedent as a reason for adopting a particular course of action. A passage carrying at least one of these markers was tagged, and the frequency of tagging was then compared across Summit statements from the 1990s, the 2000s and the 2010s, which is how the change over three decades reported in the results was established. In the second round the initial tags were grouped into the broader categories set out below, and passages that fitted none of the categories were revisited rather than discarded. All coding was carried out manually, and the full tag list and coding frame form part of the dataset deposited on figshare.

In order to achieve the strength and dependability of the answers, a number of measures were put in place. As advised by Sugiyono (2018), data triangulation was used to check the information in various documents and literature. Peer debriefing was also used. This took the form of informal discussion with two colleagues working on Southeast Asian regional politics, who read the draft coding categories and commented on them. It was not a structured expert panel, no formal interviews were conducted, and the claim is limited accordingly. The abstract has been amended so that “expert consultation” is not overstated. The practice of reflexivity was also applied, in which a researcher kept on reflecting about his/her biases and how they could affect the interpretation of data ( Creswell, 2014).

Although this study had a very rigorous methodology, this research has various limitations. Main aspects of these include reliance on secondary data material, which might not completely reflect the dynamic and changing nature of the ASEAN diplomacy. Moreover, the results of the present research can be restrictive in terms of generalizability, as it was done in the context of ASEAN and due to the peculiarities of Southeast Asia. According to Sugiyono (2018), a qualitative study is always time and place-specific, and its results tend to be local to the location of the research.

The qualitative research design that was used in the current study offers a strong platform through which the ASEAN Way and its impact on the identity of the region and peace can be explored. This research allows us to have a comprehensive picture of how the ASEAN diplomatic practice has resulted in the stability and cohesion of southeast Asia by integrating a combination of document analysis, literature and thematic analysis. The methodological rigor of the research takes into account the limitations of qualitative research, i.e., the possibility of triangulating the results and consulting the experts, ensuring the credibility of the results obtained.

## Result and discussion

### Definition and concept of the ASEAN way

The ASEAN Way, a major trademark of the ASEAN is a distinct diplomatic and procedural approach that the association has employed since its inception in 1967. The agreement-making, absence of interference of the domestic affairs of the member states and unofficial diplomacy are all believed to be the pillars of the Southeast Asian dispute resolution and cooperation in the region. These ideals emphasize the importance of consultation and respect amongst member states so that ASEAN is able to solve the issues relating to the region by diplomacy and not by conflict. Subsequently, the ASEAN Way has been considered as a significant instrument in facilitating stability and trust among the various political regimes of the Southeast Asia ( Buszynski, 1997).

The necessity to manage the diverse political, economic, and cultural orientations of the ASEAN member states and foster the growth of regional cooperation gave rise to the ASEAN Way. Beverly Loke (2005) suggests that the ASEAN Way which is characteristic of most cultural treatment of the management of international security that is instilled in the minds of ASEAN policy makers, sums up the behavioral and procedural forms which have been bureaucratized within ASEAN to thwart and handle conflicts. Although this strategy has been achieved through various challenges caused by the conflicting national or national interests as well as external pressure, it has played a significant role in the preservation of peace and security in the region.

One of the key tenets of the ASEAN Way is non-interference, which member nations have rigorously adhered to. This rule guarantees every country sovereignty and autonomy in its domestic affairs, which also contributes to the respect and trust of the ASEAN members for one another ( Tekunan, 2014). The principle was originally developed to prevent conflicts and decrease tensions amongst newly independent Southeast Asian republics. It also contributes to diplomatic unity by encouraging communication and consultation instead of direct engagement in domestic political affairs. The non-interference principle is regarded as an asset and a drawback of the ASEAN Way. Even though it has been significant in safeguarding peace among the member states, it has been faulted on failing to take more decisive action on those issues that would require collective action such as the violation of human rights and threats across national boundaries.

The concept of the ASEAN Way is also highly connected with the political and historical situation of Southeast Asia. Katsumata (2003) argues that the ASEAN Way was formulated due to the interest of the Southeast Asian countries in redefining international standards, such as non-interference in the well-defined environment in the region. Political issues that have had an impact on this restoration project are the urge to preserve domestic peace and the issue of state sovereignty. These issues gave birth to the ASEAN Way which is perceived as a viable solution to regional politics which relies on the principles of autonomy and non-interference as the means of protection against external invasion and internal turmoil.

The ASEAN Way has not been without its problems, particularly when it came to attempting to adjust to new geopolitical and security demands to do so despite its anchor value in the diplomacy culture of ASEAN. In one instance, Haacke (1999) notes that the concept of flexing engagement by some ASEAN members is an in-house problem to the ASEAN Way that suggests a more activist-oriented approach to the region, which may require the assessment of the rigorous compliance. This debate emerged as ASEAN was facing increasing regional challenges such as political crisis and transnational security challenges which required concerted efforts. It also suggests the increasing awareness that there might be a need to change the conventional diplomatic practices by ASEAN to remain relevant in the changing regional environment. This elicits a dilemma in adhering to the set standards and new realities to conform, thus depicting the dynamic characteristics of the ASEAN Way and its capacity to evolve. This discussion arose when ASEAN confronted growing regional difficulties, including political crises and transnational security concerns, which necessitated collective action. It indicates a growing realization that ASEAN’s traditional diplomatic tactics may need to evolve in order to stay effective in a changing regional environment. This highlights the conflict between adhering to established norms and adapting to new realities, demonstrating the dynamic nature of the ASEAN Way and its capacity for evolution.

Even while the ASEAN Way has historically promoted regional stability through non-interference and consultation, current geopolitical changes have put these long-standing ideals to the test. The limitations of consensus-based diplomacy in quickly addressing pressing regional challenges have been made clear by issues like the South China Sea disputes, the political crisis in Myanmar, global environmental problems, and regional humanitarian issues. As a result, the question of whether ASEAN’s institutional framework needs more adaptable and rule-based processes to preserve regional relevance and efficacy has become a major topic of scholarly discussion.

The ASEAN Way is nevertheless crucial to preserving regional security and diplomatic participation in Southeast Asia in spite of these institutional constraints. Additionally, the ASEAN Way has been crucial in helping ASEAN take the lead in regional security cooperation, particularly through the ASEAN Defence Ministers Meeting (ADMM) and ASEAN Regional Forum (ARF). These platforms, which are based on the principles of the ASEAN Way, have made it possible for ASEAN to engage the main external powers without diminishing its importance in the regional security framework ( Heng, 2014). The ASEAN Way has also played an instrumental role in assisting ASEAN to be at the forefront in cooperating on regional security, especially ASEAN Defence Ministers Meeting (ADMM) and ASEAN Regional Forum (ARF). It is through these platforms that are founded on the principles of the ASEAN Way that the ASEAN has been enabled to involve the primary external powers without downgrading its role in the regional security structure ( Heng, 2014). Nonetheless, these engagements are inherently constrained by the same norms via which these engagements are executed, especially, consensus-based decision-making process, which is prone to deliver stalling and watered-down conclusiveness during crisis periods.

It is true that ASEAN has succeeded in ensuring peace and security in Southeast Asia due to its own approach to regional diplomacy, which is called the ASEAN Way. The ASEAN Way is facing challenge due to the necessity to adjust to the new regional and global processes despite its success in promoting the development of collaboration and avoiding conflicts among the member states. As ASEAN strives to overcome them, the ASEAN Way will probably remain extolled as a source of peace as much as it will be criticized to fail in meeting more and more complicated and global issues.

Another important aspect of the ASEAN Way is that it focuses on communication and consensus as some of the key regional cooperation tools. Rather than formal legal mechanisms or force, ASEAN is promoting amongst member countries consultation and creating a gradual trust. This is the diplomacy that has helped the region to be stable, even with the various political systems, cultures, and historical challenges. Leszek Buszynski (1997) argues that the cooperative approach of ASEAN has helped the member states to settle differences between each other without necessarily engaging in a dispute. Equally, Juergen Haacke (1999) notes that the ASEAN Way depicts a common diplomatic culture which appreciates harmony, mutual respect, joint effort among the regions when addressing political and security issues.

## Discussion

### ASEAN Regional identity

A regional identity in ASEAN is a complex and emerging phenomenon, a peculiarity of which is dictated by a strange mix of the past, cultural diversity, and political expediency. During the formation of the ASEAN in 1967, it has been struggling to make sure that the member states share a common identity that supersedes the massive differences within the member states. The ASEAN Way’s tenets of non-interference, consensus-building, and respect for sovereignty are fundamental to this identity.

The ASEAN regional identity has been established through the principles of common norms and values that are meant to promote peace and stability in Southeast Asia. Rumelili (2007) has indicated that ASEAN has managed to promote the development of a collective identity of Southeast Asian states on the basis of these norms that have led to a high degree of conflict among a group of diverse states with a history of conflict. Such a sense of collective identity is not just a creation of unified cultural and historical experiences but also a political instrument that is aimed at strengthening corporate cooperation and integration in the region.

The history of the development of the regional identity in ASEAN can be dated back to the period when the major agenda of this organization was to temper inter-state relations and to avoid conflict. According to Nesadurai (2008), the initial emphasis ASEAN had was to stabilize the inter-state relations, which provided the foundation of wider regional identity. This identity has, over the years, expanded to include security cooperation as well as economic and socio-cultural integration, which can be seen through such missions as the ASEAN Community and ASEAN Charter.

The adoption of the Charter of ASEAN in 2008 is a great stepping stone in the process of forming the regional identity of ASEAN. It codifies the values and principles guiding the ASEAN Community and preconditions the further integration in many aspects, among which are politics, security, and economics ( Lin, 2010). The adoption of the Charter is the way ASEAN commits itself to a rules-based organization, which respects its collaborative norms in the face of globalization and regionalization.

Irrespective of these attempts, the process of creating a regional identity in ASEAN has not been free of obstacles. Cultural diversity of the member states of ASEAN, language, political systems, etc, is a major challenge facing the establishment of a strong regional identity. According to Koh (2007), the attempts of ASEAN to establish a socio-cultural community and ASEAN identity have to be balanced between the establishment of the regional identity and the acknowledgement of individual identities of the member states. This strike is essential in the process of making sure that the regional integration process does not undermine the cultural and political peculiarities that distinguish each member state.

Moreover, the capability of ASEAN as a regional identity is also doubtful when it comes to its relations with outside forces and its contribution to the overall East Asian regionalism. Though ASEAN has gone further towards the establishment of a common regional identity, as it is contended by Jones and Smith (2007), the reality of international relations and the role of external players still weigh down on the cohesiveness of this identity. As had been the case with the ASEAN plus Three (APT) process, there are complexities of extending the regional identity of ASEAN to the Northeast Asian nations, such as China, Japan, and South Korea, whereby divergent interests and identities are involved ( Terada, 2003).

It is through consensus-based decision-making that ASEAN was able to sustain peace and security in the region. At the same time, however, this process made it difficult for the organization to respond immediately to issues relating to politics and human welfare. Consequently, decision-making strategies that can boost the effectiveness of ASEAN as an institution while ensuring regional cooperation continue to be explored today. According to Limsiritong and Sookhakich (2025), Qualified Majority Voting (QMV) can enable ASEAN to effectively address its immediate regional issues, specifically in terms of protecting the welfare of its citizens. Through QMV, ASEAN will be able to make collective decisions without having the unanimous approval of all member states, contrary to the traditional consensus system. More debates regarding the possibility of ASEAN gradually moving towards flexible and rules-based governance structures are evident from these propositions.

The challenges of expansion and membership also have an impact on the development of ASEAN’s regional identity. Timor-Leste serves as an example of how political, organizational, and economic factors continue to be used to negotiate and redefine regional identity within ASEAN. Limsiritong and Sookhakich (2024) claim that Timor-Leste’s accession process exposed structural and legal barriers within the ASEAN Charter, especially with regard to administrative capability, economic preparedness, and agreement among current member states. The controversy surrounding Timor-Leste’s membership serves as an example of how institutional congruence and political agreement influence ASEAN regional identity in addition to geographic affiliation. This story demonstrates how ASEAN identity is developing in the face of shifting regional circumstances.

The case for qualified majority voting is not one-sided, and the objection to it deserves as much weight as the argument in its favour. The ASEAN Way rests on the understanding that no member will be outvoted on a matter it considers vital, and it is that understanding, rather than any procedural rule, which persuaded states with recent histories of mutual suspicion to keep meeting at all. Removing it would remove the assurance that brings smaller members to the table, and the trust the ASEAN Way was designed to build could be spent in the attempt to make ASEAN faster. There is a legal obstacle as well. Limsiritong and Sookhakich (2023) point out that the Charter nowhere defines what counts as a serious breach and leaves its interpretation to a Summit that itself operates by consensus, so that any move towards majority voting would first require the agreement of the members most likely to be outvoted under it. On the evidence available, qualified majority voting is better read as an aspiration than as a reform ASEAN is presently in a position to adopt.

The regional identity of ASEAN is a dynamic and multifaceted construct that has been at the core of the activities of the organization in ensuring that the region of Southeast Asia is at peace, stable, and cooperative. Although there has been a lot of progress concerning the promotion of a collective identity, concerns related to internal diversity and the external forces seem to remind ASEAN that it should always evolve and enhance its regional identity. The ASEAN Charter and the ASEAN Civic efforts are some of these steps in this direction, but it is the capacity of ASEAN to reconcile the alterations in the interests of the member states with the overall objective of regional integration that will determine whether these efforts will succeed.

A further question is whose identity is being described. The evidence assembled in this study comes almost entirely from officials, and it shows a settled and repeated regional vocabulary among foreign ministries, permanent representatives and Secretariat staff. It does not show the same thing among the wider public, where survey work has repeatedly found low awareness of ASEAN and limited attachment to it. The identity produced by the ASEAN Way is therefore best described as elite-level and institutionally maintained, and the claims made in this paper are confined to that level. This also raises the possibility that the ASEAN Way serves two purposes at the same time. It does build shared expectations among the officials who work within it, but it also shields governments from outside scrutiny of their domestic conduct, and the non-interference principle is valuable to them for precisely that reason. Both readings fit the record, and this study does not claim that the first cancels out the second.

Table 1 describes the institutional procedures employed by the ASEAN diplomacy to maintain peace. Member states participate in communication, preventative diplomacy, and cooperation via forums such as the ASEAN Summit, the ASEAN Regional Forum (ARF), and the ASEAN Political-Security Community (APSC). These procedures minimize tensions, resolve conflicts peacefully, and strengthen stability in Southeast Asia, illustrating how the ASEAN Way promotes long-term regional peace.

**Table 1.** ASEAN Institutional mechanisms supporting regional peace.

ASEAN mechanism	Function	Contribution to regional peace
ARF	Stage for discourse on safety matters	Promotes confidence-building among regional actors
APSC	Framework for political and security cooperation	Encourages collective approaches to regional stability
TAC	Establishes principles for peaceful relations	Institutionalizes peaceful conflict resolution
ASEAN Summit	Annual meeting of ASEAN leaders	Strengthens policy coordination and regional unity
ASEAN Defence Ministers’ Meeting (ADMM)	Dialogue among defence ministers	Enhances military cooperation and security collaboration

**Figure 1.**
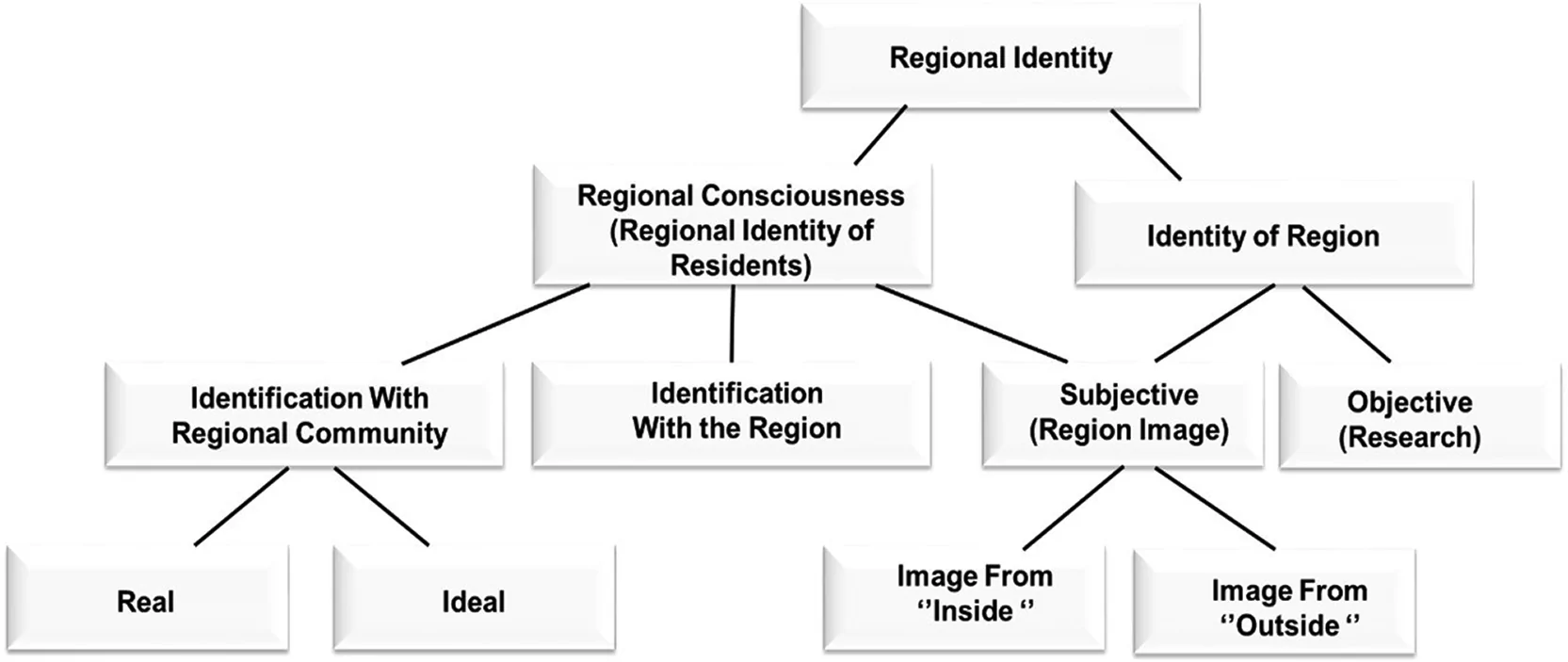
Conceptual framework of regional identity formation.

Figure 1 demonstrates the concept of the regional security and diplomatic organization of ASEAN which connects the member states of ASEAN and other foreign countries. It highlights the fact that ASEAN is the most important diplomatic center in the Asia-Pacific region. Through the ASEAN regional forum (ARF), ASEAN plus Three (APT) and the East Asia Summit (EAS) institutional forums, ASEAN is assisting the conversation and collaboration with relevant regional and global communities including China, Japan, South Korea and the US, among others. These models encourage proactive diplomacy, dispute resolution, and confidence-building. As a result, ASEAN uses diplomatic communication and multilateral cooperation to bring about peace and stability in the area Paasi (1986).

### The role of the ASEAN way in regional peace

The so-called ASEAN Way, which avoids the use of force, consensus-building, and casual mediation, has been judged essential to Southeast Asia’s peace and security. In spite of the complicated political and cultural environment of the area, where it was founded in 1967, ASEAN has proven to be one of the most positive partners of the region in terms of conflict management and, subsequently, the advancement of the law and order.

The ASEAN peace is intensely embedded in the ideals of the organization especially the principle of non-interference. Since every state considers the sovereignty of other states and does not interfere with their internal affairs, this principle has turned out to be very crucial in avoiding conflicts among the ASEAN member states. According to Tekunan (2014), the principle of non-intervention that exists in the ASEAN Way has created an environment of mutual respect and trust between its countries of the region, which has played an essential role in ensuring peace in the region. This has enabled ASEAN to move through the high tensions without necessarily engaging in conflict which ensures that the peace in the region is maintained.

Moreover, consensus based decision-making has been one of the primary reasons, which has simultaneously resulted in the absence of conflicts and improved coordination among the member states of the ASEAN Way due to its heterogeneous composition. Beverly Loke (2005) argues that despite some of the situations where such a strategy may lead to delay in decision-making, it has been good to ensure that the member states feel included and their concerns are heard. This participatory style has helped in preventing worsening of conflict situations since decisions are made when there is a broad agreement among the members hence reducing probability of action being pursued on personal terms which can lead to conflict.

Another way the ASEAN Way has contributed to peace in the region is how ASEAN has been managing external war and other security issues. As an example, the reaction of ASEAN to the Cambodian crisis in the late 1970s and 1980s explains the success of the ASEAN Way in addressing regional security problems. Kyudeug Hwang (2019) explains the way ASEAN, by applying the principles of ASEAN Way, managed to contribute to the solution to the Cambodian conflict in a substantial manner, thus cementing its peace and stability agenda in Southeast Asia. The success is highly referred to as a prime example of how ASEAN can control the security in the region with its own diplomatic system.

Although the ASEAN Way has historically helped manage conflicts and promote regional peace, current geopolitical and transnational security issues have made the institutional shortcomings of its long-standing diplomatic ideals more apparent. Heng (2014) notes that the ASEAN Way has had some success in ensuring peace in the region; however, it encounters many challenges when it comes to external issues like the aggressiveness of China in the South China Sea. The consensus form of approach can cause inactivity or poor reactions, especially when member states have conflicting interests or when outside forces are highly influential.

Nevertheless, the ASEAN Way remains a key framework for regional collaboration and healing in Southeast Asia in spite of these institutional and geopolitical obstacles. Despite these obstacles, the ASEAN Way has remained essential to maintaining peace in Southeast Asia. Despite being at the center of the regional security structural composition, its principles have allowed ASEAN to include the major external powers in regional security debates, notably the ASEAN Regional Forum (ARF). As Husain (2017) argues, the fact that ASEAN has been able to promote the positive and negative peace, i.e. to minimize the occurrence of direct conflict and increase the levels of cooperative relationships, proves that ASEAN Way remains topical and applicable in the context of the changing security environment of Southeast Asia.

The ASEAN Way has played a crucial role in promoting and maintaining peace in the region of Southeast Asia. Due to the non-interference, consensus, and informal diplomacy principles, ASEAN has been in a position to prevent conflicts in the region, facilitate cooperation, and solve complex security problems. Although the shortcomings of the ASEAN Way still exist within the framework of emerging problems and transnational threats, it is still a significant paradigm in the cycle of providing peace and security to Southeast Asia.

Setting four cases side by side makes clearer when the ASEAN Way opens channels and when it produces deadlock. In the Cambodian conflict ASEAN held a common position for more than a decade and used its collective weight in international forums, largely because every member read the Vietnamese intervention as the same kind of threat. In the South China Sea that shared reading does not exist, since members with claims and members without them have different stakes, and the result has been the repeated failure to agree a joint position and a Code of Conduct still unfinished after two decades of negotiation. The Myanmar crisis shows the limit more sharply again: the Five-Point Consensus was agreed but never enforced, because non-interference gave the authorities in Naypyidaw grounds to disregard it and gave ASEAN no means of response beyond exclusion from summits. The Rohingya case shows the same pattern in its clearest form, with the matter handled as a humanitarian and refugee problem rather than as one of accountability, which allowed cooperation on relief while leaving the cause untouched. The pattern across the four cases is consistent. Where member interests converge, the ASEAN Way supplies a workable framework for common action; where they diverge, consensus and non-interference convert disagreement into institutional paralysis. The variable is not the strength of the norms but the distribution of interests among those bound by them.

As seen in Table 2, ASEAN diplomatic rules build up a similar identity among member states. The principles of agreement, non-interference and informal diplomacy as exemplified in the ASEAN Way promote a culture of cooperation in politics and countries with divergent political systems and cultures can work together. This saw ASEAN gradually forge a regional identity based on trust, dialogue and respect.

**Table 2.** Core principles of the ASEAN way and their role in regional identity formation.

ASEAN way principle	Description	Contribution to regional identity
Consensus-based decision making	Decisions are made through agreement among all member states rather than voting	Promotes unity and collective ownership of regional policies
Non-interference	Member states avoid intervening in the internal affairs of others	Builds mutual respect and trust among diverse political systems
Informal diplomacy	Preference for dialogue, consultation, and informal negotiation	Encourages cooperative culture and long-term diplomatic relationships
Quiet diplomacy	Disputes are handled through behind-the-scenes negotiations	Prevents escalation of conflicts and preserves regional harmony
Respect for sovereignty	Each state’s political independence and territorial integrity are recognized	Strengthens shared identity while respecting national diversity

**Figure 2.**
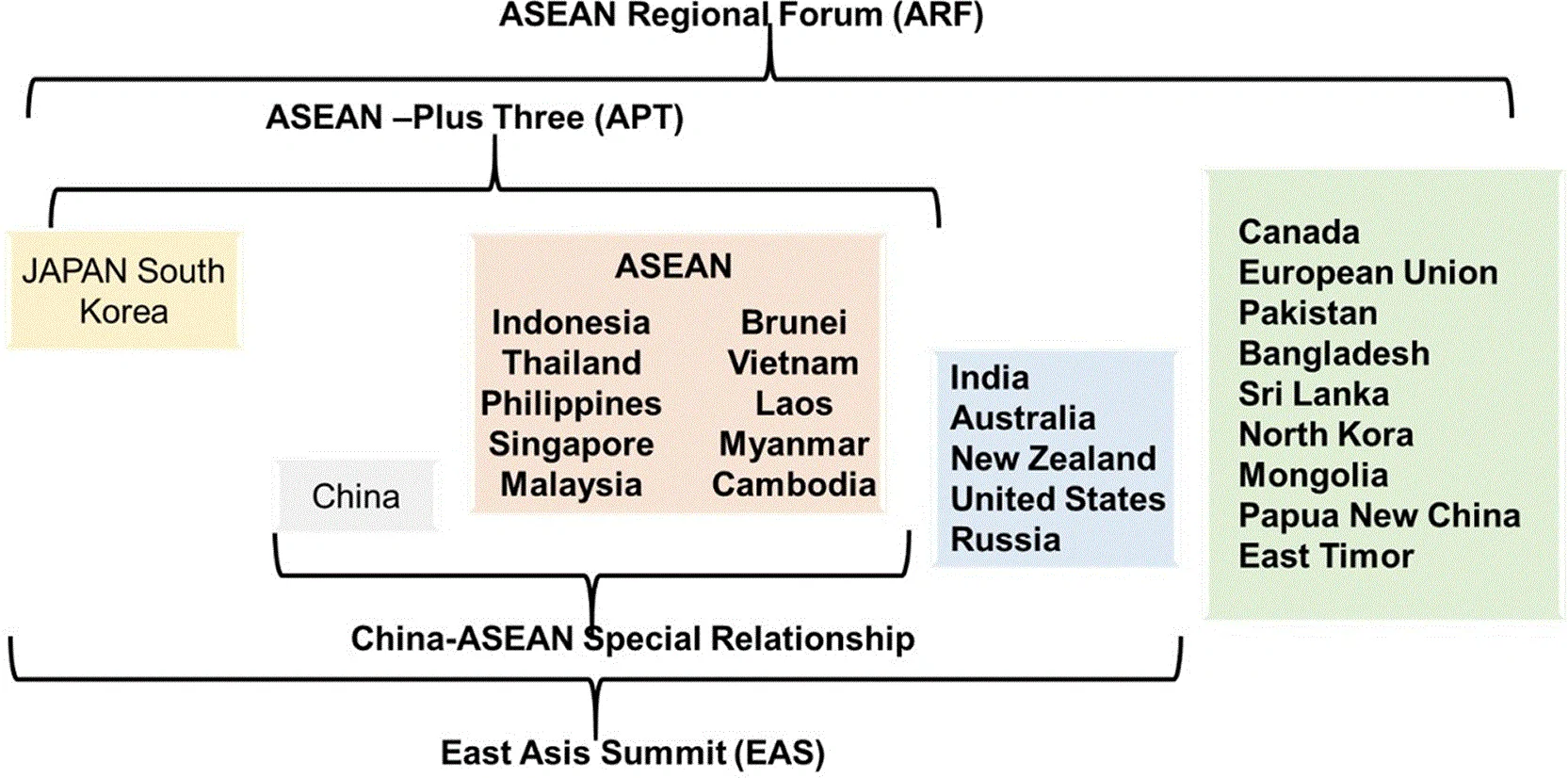
Structure of ASEAN-centered regional security and diplomatic forums.

Figure 2 shows the conceptual framework for regional identity building inside a regional organization, such as ASEAN. According to the theory, regional identity comes from the interaction of two fundamental dimensions: regional consciousness and the region’s identity. Regional awareness refers to how individuals, groups, and states understand themselves as part of a larger regional community. In contrast, the region’s identity describes how the territory is characterized by both internal perceptions (“image from inside”) and external perspectives (“image from outside”). These interwoven factors help to build a collective sense of belonging, shared standards, and common values, all of which contribute to the creation of a stronger regional identity ( Sokla, 2019).

### Challenges to the ASEAN way

Over the past 50 years, ASEAN has been successful in maintaining peace and fostering collaboration in Southeast Asia thanks in large part to consensus, non-interference, and informal diplomacy the cornerstones of the ASEAN Way. This special arrangement has seen ASEAN scale through the extensive and intricate political, cultural, and economic environment of the region to become one of the most durable regional organisations outside Europe. Durability, however, is not the same as effectiveness. ASEAN has a strong record in avoiding war between its members and in keeping dialogue going, but its record in settling disputes, protecting human rights and enforcing its own decisions during crises remains contested, and the claim made here is limited to the first of these.

Nevertheless, with the ongoing development of the region and the world, the ASEAN Way has to struggle with great challenges. The values that used to have guaranteed unity and stability are now viewed as a limitation to dealing with contemporary, transnational challenges that are manifested in the form of human rights, economic integration, and non-traditional security dangers. The consensus principle can contribute to inefficiency and slowness, and the principle of non-interference usually delays the ASEAN capability to efficiently handle internal crises inside member states. From the outset, scholarship has tended to focus on the obstacles facing the application of universal human rights norms in the region ( Aguirre and Pietropaoli, 2012).

These challenges notwithstanding, the ASEAN Way continues to be a key tool of regional diplomacy in Southeast Asia. Its flexibility and focus on communication and collaboration are still useful in dealing with the complex procedures of the region. But to survive and remain pertinent in the twenty-first century, ASEAN can have to reconsider its initial concepts to become more decisive and acting as one without acting in opposition to the interest of its member states. Non-interference applies in the economic sphere as well as the political one, and it has caused similar difficulties there. Jong (2010) argues that closer integration between the ASEAN Free Trade Area (AFTA) and the ASEAN Surveillance Process (ASP) would strengthen regional economic coordination, but that this depends on members accepting a degree of mutual scrutiny that non-interference has so far discouraged. 

Finding a balance between the core values that have historically formed the basis of the ASEAN success and adjusting to the requirements of a more interconnected and complex global context is likely to become the main staple of the future of the ASEAN Way. In so doing, the ASEAN will remain on the frontline of achieving prosperity, peace and stability in the Southeast Asian region and beyond.

## Conclusion

Over the past 50 years, ASEAN has successfully ensured peace and cooperative efforts in South East Asia due to the ASEAN Way, which is based on the principles of consensus, non-interference, and informal diplomacy. This distinctive strategy has enabled ASEAN to pass through the complicated and multicultural political, cultural, and economic environment of the area and to become one of the longest-surviving regional organisations outside Europe, although survival should not be read as evidence of effectiveness on every measure.

Nevertheless, ASEAN Way encounters very serious challenges as the world and the region are evolving. The ideals that previously ensured unity and stability are now seen as constraints in the quest to achieve modern global issues such as human rights of the human being, economic integration as well as the unconventional security threats. The reliance on consensus may result in inefficiencies and delayed responses, even if the concept of non-interference may frequently restrict ASEAN’s capacity to handle domestic problems within member nations.

These challenges notwithstanding, The ASEAN Way has remained a key model in the diplomacy of the Southeast Asian region. Its flexibility and emphasis on communication and cooperation remain useful in managing the complexity of the region. Nevertheless, to remain relevant and effective in the twenty-first century, ASEAN may have to alter its approach to the situation, perhaps by redefining its principles to allow it to take more decisive steps and be more integrated without compromising the diverse interests of its member states.

The capacity of the ASEAN Way to balance both the need to maintain the main principles underpinning the success of ASEAN and the adaptation to the requirements of a more interconnected and complex globalized environment is likely to define the survival of the ASEAN Way. This way, ASEAN is able to retain its central position in promoting prosperity, peace and security in southeast Asia and beyond.

Rather than close with a general call for adaptation, three specific steps follow from this analysis. First, ASEAN could retain consensus for matters touching sovereignty while allowing decisions on humanitarian access, disaster response and public health emergencies to be taken by a defined majority. This would leave intact the principle that protects members on questions they regard as vital, while removing the veto from the narrow class of cases in which delay carries a human cost. Second, the undefined term “serious breach” in the Charter could be given content through an agreed list of triggers, so that the question of whether a breach has occurred is settled before a crisis rather than negotiated during one. Third, the Secretariat could be given a standing mandate to publish its own assessment of member compliance with agreed commitments. Publication compels nobody and requires no reopening of the Charter, but it removes the silence that non-interference currently supplies. None of the three abolishes the ASEAN Way. Each narrows the range of cases in which it produces paralysis, which is the more realistic path for an organisation whose members will not accept a transfer of authority.

## Limitations of the study

Despite the fact that this piece of work makes good contributions to the development of knowledge on how the ASEAN Way can contribute to the establishment of regional identity and regional peacekeeping in Southeast Asia, there are several limitations that should be taken into consideration and could influence the depth of the conclusions and generalizability. Firstly, the research paper relies on the secondary sources of data, including academic literature, official sources of the ASEAN, and case studies. Although these sources are valuable in terms of qualitative information, they might not be a comprehensive reflection of the latest trends and subtle views of the important stakeholders in ASEAN. Moreover, the use of publicly available documents might restrict the level of analysis, especially in the internal dynamics and decision-making process in ASEAN.

Second, the emphasis on qualitative analysis, though suitable to study the intricate social and political phenomena, is intrinsically associated with the inability to measure the effect of the ASEAN Way on the regional performance. This qualitative design offers a detailed approach but does not give a chance of statistical generalization as would have been the case in a mixed methods study, which would use quantitative data.

Third, the study focuses on a conceptual and historical analysis of the ASEAN Way and its significance for peace and regional identity. As a result, when applied to specific and contemporary circumstances, it does not fully address the real-life problems and responses of the member nations in the ASEAN manner. Future research can be more empirical in nature, involving interviews with officials and practitioners, in order to gain firsthand knowledge of the difficulties and efficacy of the ASEAN Way in the current geopolitical situation.

Lastly, the paper also has limitations in terms of its geographical scope because it is based in Southeast Asia, and this is not entirely representative of the external factors and global trends that are increasingly affecting the region. Although the paper also brings up the issue of potential external actors (China and the US) and the impact they have on the ASEAN Way, a further discussion of the impact of global geopolitical changes on the ASEAN Way can yield a more comprehensive view of whether it is viable or not.

To sum up, although this study is a valuable addition to the scholarly discussion on ASEAN and its peculiarities of the regional diplomacy, these shortcomings indicate that future research that will fill these voids, possibly due to a more varied methodology and a wider geographic focus, is necessary.

## Data Availability

Figshare: Crafting Regional Identity and Peace through the ASEAN Way in Diplomacy. https://doi.org/10.6084/m9.figshare.31797496.v1 [ Akbar Hashemi Rafsanjani et al., 2026]. The project contains the following underlying data: Qualitative dataset (ASEAN diplomatic documents and thematic analysis codes used in the study). Data are available under the terms of the Creative Commons Attribution 4.0 International license (CC-BY 4.0).
