# Supplementary material for: Crafting Regional Identity and Peace through the ASEAN Way in Diplomacy
Source: F1000Res. 2026 Aug 14;15:621. Originally published 2026 Apr 27. [Version 3] doi: 10.12688/f1000research.179376.3 (PMC13560795; doi:10.12688/f1000research.179376.3)
Supplement: Peer review history

# Reviewer report: Reviewer response for version 3

**DOI:** 10.5256/f1000research.208241.r512609

**Published:** 2026-08-26

**Authors:** Muhammad Jahanzaib

**Affiliations:** 1 Diamanium Thinkers, Islamabad, Pakistan

**Summary of the article**

 This qualitative research article analyses the “ASEAN Way” (consensus-building, non-interference, and informal dialogue) as a distinctive diplomatic approach. Drawing on secondary sources - official ASEAN documents, academic literature, and case studies - and employing thematic analysis (with data triangulation and informal peer debriefing/expert consultation), the authors argue that the ASEAN Way has contributed to regional peace by fostering mutual trust, dialogue, and conflict avoidance, while also supporting the formation of a shared regional identity. They simultaneously note its limitations in addressing complex transnational challenges and urgent geopolitical issues arising from consensus-based decision-making and call for adaptive evolution of the principles while preserving core values.

*Answers to the specific questions for Research Articles*

1. **Is the work clearly and accurately presented and does it cite the current literature?**  Yes. The abstract and overall framing present the topic clearly and accurately. The core concepts of the ASEAN Way are standard and well-established in the literature (Acharya, Haacke, etc.). As this is version 3 of a revised manuscript, the literature review should fully engage recent scholarship on ASEAN centrality, identity construction, responses to Myanmar, South China Sea dynamics, and great-power rivalry. Any remaining gaps in currency or depth of engagement with post-2020 literature should be closed.
2. **Is the study design appropriate and does the work have academic merit?** Yes. A qualitative design based on secondary-data thematic analysis is appropriate for an interpretive study of diplomatic norms, identity formation, and peace contributions in International Relations / Southeast Asian studies. The topic has clear academic merit: the ASEAN Way remains a central (and contested) concept in regionalism debates, and examining its dual role in identity and peace is worthwhile.
3. **Are sufficient details of methods and analysis provided to allow replication by others?** Partially. The abstract describes a thematic analysis of official documents, literature, and case studies, plus triangulation and informal peer debriefing/expert consultation. For full replicability, the methods section should specify: the exact document corpus and selection criteria, coding procedure (inductive/deductive, software if any), how themes were derived and validated, the precise case studies examined, and how expert consultation was conducted. Greater transparency here would strengthen the work.
4. **If applicable, is the statistical analysis and its interpretation appropriate?** Not applicable (qualitative study).
5. **Are all the source data underlying the results available to ensure full reproducibility?** Secondary sources are cited; full reproducibility depends on clear, complete referencing of all ASEAN documents, literature, and any other materials used. Primary interview data (if any expert consultations produced formal notes) should be deposited or described in sufficient detail where ethically possible. No original quantitative datasets appear to be involved.
6. **Are the conclusions drawn adequately supported by the results?** Yes, at a general level. The findings that the ASEAN Way has supported trust, dialogue, conflict avoidance, and a degree of shared identity, while facing limitations with transnational/geopolitical urgency, align with the described results and with the broader scholarly consensus. Stronger linkage between specific themes/evidence and the more forward-looking claims about necessary adaptation would further reinforce the conclusions.

Is the work clearly and accurately presented and does it cite the current literature?

Yes

If applicable, is the statistical analysis and its interpretation appropriate?

Not applicable

Are all the source data underlying the results available to ensure full reproducibility?

Partly

Is the study design appropriate and is the work technically sound?

Yes

Are the conclusions drawn adequately supported by the results?

Yes

Are sufficient details of methods and analysis provided to allow replication by others?

Partly

Reviewer Expertise:

International Relations, Geo-Economics, Global Capital Markets, Geo-Politics

I confirm that I have read this submission and believe that I have an appropriate level of expertise to confirm that it is of an acceptable scientific standard.

# Reviewer report: Reviewer response for version 3

**DOI:** 10.5256/f1000research.208241.r511147

**Published:** 2026-08-24

**Authors:** Mohammed Torki Bani Salameh

**Affiliations:** 1 Political and International Studies Department, Yarmouk University, Irbid, Jordan

I have carefully reviewed the revised version of the article, “Crafting Regional Identity and Peace through the ASEAN Way in Diplomacy,” together with the authors’ responses and amendments.

 I am satisfied that the authors have adequately addressed my previous comments and that the revisions have substantially strengthened the manuscript’s conceptual clarity, methodological transparency, comparative analysis, and overall academic contribution.

 I therefore accept the revisions and wish to amend my previous status from “Approved with reservations” to “Approved.” I recommend the article for acceptance and indexing in its revised form.

Is the work clearly and accurately presented and does it cite the current literature?

Yes

If applicable, is the statistical analysis and its interpretation appropriate?

Not applicable

Are all the source data underlying the results available to ensure full reproducibility?

Partly

Is the study design appropriate and is the work technically sound?

Yes

Are the conclusions drawn adequately supported by the results?

Yes

Are sufficient details of methods and analysis provided to allow replication by others?

Yes

Reviewer Expertise:

NA

I confirm that I have read this submission and believe that I have an appropriate level of expertise to confirm that it is of an acceptable scientific standard.

# Reviewer report: Reviewer response for version 3

**DOI:** 10.5256/f1000research.208241.r511146

**Published:** 2026-08-18

**Authors:** Kittisak Wongmahesak

**Affiliations:** 1 North Bangkok University, Bangkok, Bangkok, Thailand

The manuscript addresses an important and timely question: whether and how the ASEAN Way contributes to regional identity formation and peace. Version 3 has substantively improved the manuscript by defining peace and regional identity, articulating a plausible constructivist mechanism, acknowledging alternative explanations, and incorporating a comparative discussion of Cambodia, the South China Sea, Myanmar, and the Rohingya crisis. The paper’s central proposition—that the ASEAN Way facilitates common action where member interests converge but produces paralysis where they diverge—is potentially valuable.

 However, the manuscript still falls short of the analytical, methodological, and presentational standard required for a robust international research article. The revisions add useful material, but much of the empirical discussion remains assertive and descriptive rather than systematically evidencing the proposed causal mechanism.

 Major issues that must be addressed:

 1) Align the research design with the causal claims: The article claims that repeated diplomatic practice produces shared identity, which subsequently affects state behavior and peace. Yet the evidence is primarily official ASEAN discourse and secondary literature. Such material can show institutional rhetoric, formal commitments, and elite-level vocabulary, but it cannot, by itself, establish that identity was internalized or that it caused particular state behavior. The authors should revise causal language throughout. They should present the ASEAN Way as a plausible contributing condition rather than demonstrate that it “significantly contributed” to peace, unless each causal link is systematically evidenced. The authors should incorporate alternative explanations—strategic restraint, regime-security interests, bilateral diplomacy, economic interdependence, external threats, and major-power influence—into the case analysis rather than acknowledging them only in the introduction.

 2) Make the four-case comparison genuinely systematic: The Cambodia, South China Sea, Myanmar, and Rohingya discussion is a promising addition but currently reads as a concise narrative comparison. The authors should provide a structured comparative table or analytical framework for each case, specifying:

 - the nature of the crisis and relevant period;

 - convergence or divergence of member-state interests;

 - ASEAN Way mechanism employed;

 - documentary evidence of consultation, consensus, restraint, or non-interference;

 - observable outcome;

 - alternative explanatory factors; and

 - the extent to which the case supports, qualifies, or challenges the central argument.

 This revision is necessary to show why Cambodia constitutes a case of coordinated action, whereas the South China Sea, Myanmar, and Rohingya cases demonstrate institutional limitation.

 3) Strengthen methodological traceability and replicability: The document-selection criteria and coding description have improved, but the analysis remains insufficiently auditable. The claim that “frequency of tagging” was compared across decades is especially important, yet the authors report no counts, document-level results, coding extracts, or comparison procedure.

 The authors should provide, in the article or deposited dataset:

 - a complete list of all 42 ASEAN documents, including title, date, institutional source, document type, and stable source location;

 - the full corpus of 26 summit/foreign-ministerial communiqués and eight ARF chair statements, or clear access information;

 - the complete coding manual, definitions, inclusion/exclusion rules, and examples of coded passages;

 - a document-by-document coding matrix;

 - the frequencies or other evidence supporting claims of change across the 1990s, 2000s, and 2010s;

 - the analytical materials linking codes to each principal finding and case conclusion;

 - clarification of whether one or multiple coders undertook coding and, if one, how interpretive consistency was managed.

 Informal peer debriefing is correctly qualified, but it cannot substitute for a transparent audit trail.

 4) Clarify the contribution and boundaries of “regional identity”: The manuscript appropriately limits its identity claim to elite officials and institutions. The authors should integrate this qualification into the title, abstract, results, and conclusion—not only insert it later in the discussion. The evidence supports an institutionally reproduced elite diplomatic identity, not a broader ASEAN regional identity among citizens.

 The authors should also distinguish more consistently among:

 - shared diplomatic vocabulary;

 - institutional socialization;

 - elite identity;

 - policy convergence; and

 - a security community.

 These are related but not interchangeable outcomes. Recurrent ASEAN language may demonstrate discursive continuity, but it does not necessarily establish policy convergence or internalized collective identity.

 5) Revise the figures and tables for accuracy, relevance, and analytical value: The figures and tables are currently a substantial weakness.

 - Figure numbering and textual cross-references are inconsistent: the text refers to Figure 1 and Figure 2 in ways that do not consistently correspond with their displayed contents.

 - Figure 1 appears to reproduce a generic regional-identity framework but is not clearly adapted to the article’s constructivist mechanism or indicators.

 - Figure 2 is conceptually and factually unclear. It conflates or inaccurately represents ASEAN-centered forums and memberships, includes unclear labels, and does not substantively support the argument.

 - Tables 1 and 2 largely restate general knowledge and make unsubstantiated causal claims, such as that particular institutions “institutionalize peaceful conflict resolution” or that norms “build trust.”

 The authors should remove generic descriptive visuals or replace them with an original analytical table showing the proposed mechanism and comparative case evidence. Carefully check all captions, numbering, sources, dates, memberships, and labels.

Is the work clearly and accurately presented and does it cite the current literature?

Partly

If applicable, is the statistical analysis and its interpretation appropriate?

Not applicable

Are all the source data underlying the results available to ensure full reproducibility?

Yes

Is the study design appropriate and is the work technically sound?

Yes

Are the conclusions drawn adequately supported by the results?

Yes

Are sufficient details of methods and analysis provided to allow replication by others?

Partly

Reviewer Expertise:

Political Science, Public & Private Management, International Political Economy, Social Research

I confirm that I have read this submission and believe that I have an appropriate level of expertise to confirm that it is of an acceptable scientific standard, however I have significant reservations, as outlined above.

# Reviewer report: Reviewer response for version 2

**DOI:** 10.5256/f1000research.202180.r498377

**Published:** 2026-08-04

**Authors:** Mohammed Torki Bani Salameh

**Affiliations:** 1 Political and International Studies Department, Yarmouk University, Irbid, Jordan

The manuscript, “Crafting Regional Identity and Peace through the ASEAN Way in Diplomacy,” examines how consensus, non-interference, informal dialogue, and consultation contribute to regional identity and peace in Southeast Asia. The topic is timely, particularly in light of ASEAN’s responses to the Myanmar crisis, the Rohingya issue, South China Sea disputes, human-rights violations, and intensifying US–China competition.

 The manuscript has a clear and potentially indexable core. Its constructivist orientation and attempt to connect diplomatic practice with identity formation are valuable. Nevertheless, several theoretical, methodological, empirical, and presentational issues should be addressed.

**Required Revisions**

**1. Clarify the theoretical contribution**

 The relationship among constructivism, diplomacy theory, and regional identity should be explained more systematically. The authors need to identify the mechanism through which repeated diplomatic practices generate a shared identity and how this identity subsequently affects state behaviour.

 The manuscript should also distinguish among preventing interstate conflict, managing disagreement, and producing a meaningful collective identity. The absence of war does not necessarily prove the existence of a security community. Alternative explanations, including regime interests, economic interdependence, strategic calculations, and external threats, should be considered.

**2. Operationalize peace and regional identity**

 “Peace,” “stability,” “conflict avoidance,” and “security cooperation” are sometimes used interchangeably. The authors should specify whether peace means the absence of interstate war, effective dispute resolution, domestic stability, human security, or positive peace.

 Regional identity should likewise be measured through observable indicators, such as common diplomatic language, institutional commitments, policy convergence, elite identification with ASEAN, and shared expectations of peaceful conduct.

**3. Improve methodological transparency**

 The methodology should report the number and types of documents examined, the period covered, inclusion and exclusion criteria, thematic coding procedures, and methods of triangulation. The authors should also explain the number, expertise, and selection of consulted experts. If expert consultation was informal or unsystematic, the claim should be qualified or removed.

 The manuscript must demonstrate how its evidence supports conclusions about identity formation and peace rather than merely reproducing ASEAN’s official discourse.

**4. Deepen the empirical analysis**

 The discussion remains largely descriptive. The argument should be tested through a structured comparison of selected cases, such as the Cambodian conflict, South China Sea disputes, Myanmar crisis, and Rohingya issue. Such comparison would identify when the ASEAN Way facilitates dialogue and when consensus and non-interference result in institutional paralysis.

**5. Balance achievements and limitations**

 Claims describing ASEAN as one of the most successful regional organizations outside Europe should be qualified. ASEAN has contributed to conflict avoidance and sustained diplomatic engagement, but its effectiveness in dispute resolution, human-rights protection, democracy, and crisis enforcement remains contested. Institutional durability should not automatically be equated with policy effectiveness.

**6. Improve structure and language**

 Repetition across the literature review, findings, discussion, and conclusion should be reduced. Substantial language editing is also required. Expressions such as “provincial character,” “regional corporations,” “learning have tended,” and “a input” should be corrected. Citations and references must follow a consistent APA format, while figure numbers, captions, and content should be carefully matched.

**Recommended Academic Literature**

 The following works would strengthen the manuscript’s theoretical and empirical foundations.

 Acharya’s security-community framework explains how consultation, socialization, restraint, and repeated interaction can generate shared expectations of peaceful conduct. It would help the authors establish criteria for determining whether ASEAN has developed a genuine security community.

 Ba conceptualizes regional identity as an evolving and contested construction rather than an automatic consequence of institutional membership. This perspective would improve the discussion of how ASEAN identity is negotiated through regional discourse, institutional practices, and interaction with external powers.

 Stubbs offers a balanced framework for comparing ASEAN proponents and sceptics. His analysis would help distinguish among institutional durability, conflict avoidance, diplomatic influence, and effective problem-solving.

 Davies challenges purely constructivist interpretations by identifying an action–identity gap between ASEAN’s formal normative commitments and the actual conduct of its members. This is particularly relevant to the manuscript’s treatment of human rights, Myanmar, and the Rohingya crisis.

 Jackson explains why Asian states frequently hedge rather than balance exclusively against or align fully with a major power. His study would strengthen the analysis of ASEAN’s strategic flexibility and its members’ divergent responses to US–China competition.

 Hu provides a focused assessment of ASEAN’s effectiveness in managing the South China Sea disputes. This work would help the authors distinguish continued dialogue from successful dispute management or meaningful changes in state behaviour.

**Comparative Literature**

 The following studies may also be integrated as comparative evidence. They should not be presented as direct evidence about ASEAN but as examples of how other regional actors employ mediation, hedging, flexible alignment, and multilevel security strategies.

 Bani Salameh and Alotaibi demonstrate how Kuwait uses neutrality, mediation, diplomatic credibility, and soft power to exercise influence despite limited material capabilities. The comparison supports the manuscript’s discussion of informal diplomacy, preventive engagement, and conflict management.

 Bani Salameh and Darabseh show how the Gulf crisis transformed Qatari–Turkish relations while weakening Gulf Cooperation Council cohesion. Their study illustrates how divergent threat perceptions and external partnerships can undermine regional institutional unity, offering a useful comparison with ASEAN’s divisions over China and the South China Sea.

 Bani Salameh and Mubiadeen examine how small Gulf states employ different combinations of neutrality, hedging, alignment, and alliance diversification. Their findings help explain why ASEAN members may share regional norms while pursuing divergent national security strategies.

 Bani Salameh’s study of Bahrain adopts a multilevel approach connecting domestic vulnerabilities, regional cooperation, and international partnerships. This framework could help explain how ASEAN members reconcile domestic priorities, regional identity, and external-power relations.

 Finally, Bani Salameh’s analysis of Kuwait demonstrates how small states use strategic hedging to maintain relations with competing powers without exclusive alignment. This offers a useful comparison with ASEAN members’ simultaneous economic engagement with China, security cooperation with the United States, and support for ASEAN-centred multilateralism.

**Final Recommendation**

 The manuscript offers a potentially valuable contribution to scholarship on ASEAN diplomacy, constructivism, regional identity, and conflict management. Subject to theoretical clarification, improved methodological transparency, deeper comparative analysis, reduced repetition, and substantive engagement with the recommended literature, I recommend **acceptance after minor revision**.

Is the work clearly and accurately presented and does it cite the current literature?

Yes

If applicable, is the statistical analysis and its interpretation appropriate?

Not applicable

Are all the source data underlying the results available to ensure full reproducibility?

Partly

Is the study design appropriate and is the work technically sound?

Yes

Are the conclusions drawn adequately supported by the results?

Yes

Are sufficient details of methods and analysis provided to allow replication by others?

Yes

Reviewer Expertise:

NA

I confirm that I have read this submission and believe that I have an appropriate level of expertise to confirm that it is of an acceptable scientific standard, however I have significant reservations, as outlined above.

## References

[1] Acharya, A. (2014). Constructing a security community in Southeast Asia: ASEAN and the problem of regional order (3rd ed.). Routledge.

[2] Ba, A. D. (2009). (Re)negotiating East and Southeast Asia: Region, regionalism, and the Association of Southeast Asian Nations. Stanford University Press.

[3] : Bahrain's Vision in the New Gulf Order. *Middle East Policy* .2023;30(1) : 10.1111/mepo.12675110-121 10.1111/mepo.12675

[4] Bani Salameh, M. T. (2024). Kuwait’s hedging strategy toward Iran and Saudi Arabia. Middle East Quarterly, 31(2), 1–11. https://www.meforum.org/middle-east-quarterly/kuwait-hedging-strategy-toward-iran-and-saudi.

[5] : Kuwait as a Regional Mediator: Small-state Diplomacy and Soft Power in Middle Eastern Conflicts. *Contemporary Review of the Middle East* .2026;13(1) : 10.1177/2347798925140050654-74 10.1177/23477989251400506

[6] : Strategic shifts: the role of the Gulf Crisis in transforming Qatari-Turkish relations (2017–2021). *Review of Economics and Political Science* .2026;11(1) : 10.1108/REPS-04-2025-003779-96 10.1108/REPS-04-2025-0037

[7] : Addressing security challenges: Strategic approaches of Arab gulf states. *International Journal of Innovative Research and Scientific Studies* .2025;8(1) : 10.53894/ijirss.v8i1.48071853-1864 10.53894/ijirss.v8i1.4807

[8] : ASEAN and human rights norms: constructivism, rational choice, and the action-identity gap. *International Relations of the Asia-Pacific* .2013;13(2) : 10.1093/irap/lct002207-231 10.1093/irap/lct002

[9] : Examining ASEAN’s effectiveness in managing South China Sea disputes. *The Pacific Review* .2023;36(1) : 10.1080/09512748.2021.1934519119-147 10.1080/09512748.2021.1934519

[10] : Power, trust, and network complexity: three logics of hedging in Asian security. *International Relations of the Asia-Pacific* .2014;14(3) : 10.1093/irap/lcu005331-356 10.1093/irap/lcu005

[11] : ASEAN sceptics versus ASEAN proponents: evaluating regional institutions. *The Pacific Review* .2019;32(6) : 10.1080/09512748.2019.1611627923-950 10.1080/09512748.2019.1611627

# Reviewer report: Reviewer response for version 2

**DOI:** 10.5256/f1000research.202180.r496642

**Published:** 2026-06-30

**Authors:** Kittisak Wongmahesak

**Affiliations:** 1 North Bangkok University, Bangkok, Bangkok, Thailand

The authors are to be commended for their responsiveness to the first round of peer review; the integration of constructivist theory and the inclusion of contemporary crises—notably Myanmar, the Rohingya refugee situation, and Timor-Leste’s accession—significantly elevate the paper’s relevance. However, to meet the elite standards of a premier international outlet like *F1000Research*, several critical analytical and structural weaknesses remain that require "final sharpening."

 The primary concern lies in the depth of theoretical synthesis. While the authors correctly identify constructivism as the foundation for regional identity formation, the application remains somewhat descriptive. A sharp academic argument must move beyond stating that norms shape behavior to explaining how specific discursive practices within the "ASEAN Way" successfully (or unsuccessfully) internalize a collective identity among elite state actors versus the general populace. The authors should critically interrogate whether the "ASEAN Way" acts as a genuine identity-builder or merely as a convenient diplomatic shield for regime security. Strengthening this theoretical bridge would transform the paper from a comprehensive summary into a leading contribution to the debate on normative regionalism.

 Regarding the sharpness of the argument on institutional reform, the discussion of Qualified Majority Voting (QMV) is a valuable addition but would benefit from a more forceful analytical stance. The authors present QMV as a potential solution to the "slowness" of consensus. Yet, they miss the opportunity to engage with the counter-argument: would QMV destroy the very "trust" that the ASEAN Way was designed to build? To achieve a high level of academic debate, the manuscript should offer a more nuanced exploration of this tension. For instance, citing the specific legal hurdles in the ASEAN Charter, as noted by Limsiritong and Sookhakich (2023) regarding "serious breaches," would provide a more grounded and critical assessment of whether institutional reform is practically viable or purely aspirational.

 Methodological transparency, while improved, still lacks the "concrete" detail required for full replicability in qualitative research. The authors state that they used thematic analysis following Braun and Clarke. Still, the connection between the raw secondary data (specific ASEAN documents) and the final "Results" patterns remains a "black box." To improve this, the authors should provide a more explicit description of their coding process. For example, how did they define the theme of "regional consciousness" across three decades of ASEAN Summit statements? A brief table in the supplementary materials, or a more detailed paragraph on the criteria for document selection and the specific nodes identified during the analysis, would greatly enhance the study's scientific rigor.

 Furthermore, the narrative cohesion between the historical analysis and contemporary geopolitical challenges could be more fluid. The transitions between the "South China Sea controversies" and the "Para diplomacy" sections feel somewhat disjointed. In a top-tier research article, every section must contribute to the overarching thesis. The authors should more explicitly link Utomo’s (2025) findings on sub-national diplomacy to the macro-level ASEAN regional identity. Does paradiplomacy strengthen or fragment the central ASEAN narrative? Resolving this would provide a more "holistic perception," as promised in the introduction.

 Finally, the manuscript requires a thorough linguistic and stylistic polish. While the "Revised" version has improved, there are still instances of repetitive phrasing and "report-like" structures that disrupt the sophisticated prose expected of international scholars. The authors should avoid excessive subheadings and ensure that the "Conclusion and Discussion" section does more than restate the findings; it should offer a "concrete proposal" for ASEAN’s evolutionary path in the 21st century. If the authors can bridge these remaining gaps—moving from description to critical synthesis and from general thematic mapping to methodological transparency—this manuscript will serve as a robust and original contribution to Southeast Asian diplomatic studies. I recommend one final round of "Major Revision" to achieve this standard.

Is the work clearly and accurately presented and does it cite the current literature?

Partly

If applicable, is the statistical analysis and its interpretation appropriate?

Not applicable

Are all the source data underlying the results available to ensure full reproducibility?

Yes

Is the study design appropriate and is the work technically sound?

Yes

Are the conclusions drawn adequately supported by the results?

Yes

Are sufficient details of methods and analysis provided to allow replication by others?

Partly

Reviewer Expertise:

Political Science, Public & Private Management, International Political Economy, Social Research

I confirm that I have read this submission and believe that I have an appropriate level of expertise to confirm that it is of an acceptable scientific standard, however I have significant reservations, as outlined above.

# Reviewer report: Reviewer response for version 1

**DOI:** 10.5256/f1000research.197881.r479965

**Published:** 2026-05-11

**Authors:** Yusa Djuyandi

**Affiliations:** 1 Universitas Padjadjaran, Bandung, West Java, Indonesia

This article have not yet discuss and show the adequate concepts and theories. Diplomacy concepts and theories together with regional identity are also important to be discussed as an integrated conceptual framework.

 Authors also need to explain the novelty from this studies by examining some relevant previous studies. Please to examine these previous studies:

 1. Unraveling Capability & Capacity of Parallel Diplomacy Governance in Indonesia: How Far Are Indonesia’s Regional Governments Ready to Go?

 2. Diplomacy, migration, and employment: The historical approach to Malaysia–Indonesia relations

 3. Paradiplomacy of the Global South: Surabaya's Approach to Global Sustainability (2016-2020).

 Reference citation must be consistent. Please only write their last name and year, eg: Loke, 2015; Pedrason, 2017, etc.

 Authors also have to explained more detail about the high tension on South China Sea, how the conflict arise between ASEAN members, China, and United States.

Is the work clearly and accurately presented and does it cite the current literature?

Partly

If applicable, is the statistical analysis and its interpretation appropriate?

Not applicable

Are all the source data underlying the results available to ensure full reproducibility?

Partly

Is the study design appropriate and is the work technically sound?

Partly

Are the conclusions drawn adequately supported by the results?

Yes

Are sufficient details of methods and analysis provided to allow replication by others?

Yes

Reviewer Expertise:

Security studies and political science

I confirm that I have read this submission and believe that I have an appropriate level of expertise to confirm that it is of an acceptable scientific standard, however I have significant reservations, as outlined above.

## References

[1] : PARADIPLOMACY OF THE GLOBAL SOUTH: SURABAYA’S APPROACH TO GLOBAL SUSTAINABILITY CHALLENGES (2016-2020). *JANUS NET e-journal of International Relation* .2025;16(nº1) : 10.26619/1647-7251.16.1.16 10.26619/1647-7251.16.1.16

[2] : Unraveling Capability and Capacity of Parallel Diplomacy Governance in Indonesia: How Far Are Indonesia’s Regional Governments Ready to Go?. *Journal of Ecohumanism* .2024;3(7) : 10.62754/joe.v3i7.42921299-1308 10.62754/joe.v3i7.4292

[3] : Diplomacy, migration, and employment: The historical approach to Malaysia–Indonesia relations. *International Journal of Population Studies* .2025;12(1) : 10.36922/IJPS025230112 10.36922/IJPS025230112

# Reviewer report: Reviewer response for version 1

**DOI:** 10.5256/f1000research.197881.r479964

**Published:** 2026-05-04

**Authors:** Kittisak Wongmahesak

**Affiliations:** 1 North Bangkok University, Bangkok, Bangkok, Thailand

The manuscript provides a comprehensive qualitative overview of the "ASEAN Way" and its role in maintaining regional stability and identity. While the research is technically sound and the thematic analysis offers a structured narrative, the article requires significant refinement in terms of theoretical depth, critical debate, and the integration of contemporary scholarship to meet the high standards of a premier international journal.

**Theoretical Depth and Literature Review:** The current literature review is primarily descriptive and relies on established concepts of non-interference and consensus. To improve the academic quality, the authors should engage with more recent debates on structural reforms within ASEAN. Specifically, the discussion on the limitations of the ASEAN Way lacks a rigorous analysis of institutional power constraints. The authors are encouraged to incorporate Limsiritong (2018) to address the ASEAN Inter-Governmental Human Rights Commission's power limitations, particularly in high-stakes cases such as the Rohingya issue. Furthermore, the argument regarding the "Myanmar crisis" and breaches of the ASEAN Charter should be bolstered by citing Limsiritong and Sookhakich (2023) to provide a more critical assessment of whether traditional principles remain legally and politically viable amid civilian violence.

**Critical Argumentation and Reform:** The article identifies the "slowness" of consensus but does not sufficiently explore alternative mechanisms that could preserve peace while enhancing decisiveness. To strengthen the "Discussion" section, the authors should analyze the transition from consensus toward more flexible decision-making models. Engaging with Limsiritong and Sookhakich (2025) would spark a focused debate on implementing Qualified Majority Voting (QMV) as a pathway to human rights reform and provide a concrete proposal for ASEAN’s evolution. Additionally, the formation of regional identity is not merely an internal process but is affected by expansion challenges. Citing Limsiritong and Sookhakich (2024) on the obstacles to Timor-Leste’s membership under the ASEAN Charter would provide a necessary contemporary case study of how regional identity is contested and redefined.

**Methodological Rigor:** While the thematic analysis is appropriate, the "Research Methods" section is currently too brief for full replication. The authors should provide more explicit details on the criteria used to select official documents and the specific coding process employed in the thematic analysis. This would enhance the transparency of how the "Result" patterns were derived from the secondary data.

**Structural and Stylistic Requirements:** The manuscript should be reorganized into five distinct sections: Introduction, Literature Review, Research Methods, Research Results, and Conclusion and Discussion. Please maintain a cohesive prose style throughout, and avoid excessive subheadings or bullet points that interrupt the narrative flow. The transitions between the historical analysis and contemporary geopolitical challenges must be made more fluid to ensure a compelling academic argument.

 By addressing these points—particularly the inclusion of recent scholarship on the ASEAN Charter and structural reform—the manuscript will move from a general descriptive study to an original and scientifically robust contribution to Southeast Asian diplomatic studies.

Is the work clearly and accurately presented and does it cite the current literature?

Partly

If applicable, is the statistical analysis and its interpretation appropriate?

Not applicable

Are all the source data underlying the results available to ensure full reproducibility?

Yes

Is the study design appropriate and is the work technically sound?

Yes

Are the conclusions drawn adequately supported by the results?

Yes

Are sufficient details of methods and analysis provided to allow replication by others?

Partly

Reviewer Expertise:

Political Science, Public & Private Management, International Political Economy, Social Research

I confirm that I have read this submission and believe that I have an appropriate level of expertise to confirm that it is of an acceptable scientific standard, however I have significant reservations, as outlined above.
